# Disulfiram/copper triggers cGAS-STING innate immunity pathway via ROS-induced DNA damage that potentiates antitumor response to PD-1 checkpoint blockade

**DOI:** 10.7150/ijbs.105575

**Published:** 2025-02-03

**Authors:** Mengmeng Yuan, Liang Shi, Yan Liu, Ke Xiang, Yan Zhang, Ye Zhou, Jianling Wang, Meiju Ji, Peng Hou

**Affiliations:** 1 Department of Endocrinology and International Joint Research Center for Tumor Precision Medicine of Shaanxi Province, The First Affiliated Hospital of Xi'an Jiaotong University, Xi'an, Shaanxi, 710061, China.; 2 Program of Environmental Toxicology, School of Public Health, China Medical University, Shenyang, Liaoning, 110122, China.; 3 Center for Translational Medicine, The First Affiliated Hospital of Xi'an Jiaotong University, Xi'an, Shaanxi, 710061, China.

**Keywords:** Disulfiram/Copper, Innate immunity, ROS, DNA damage, Cancer immunotherapy

## Abstract

Immune checkpoint blockades (ICBs) have emerged as the leading strategy for treating advanced malignancies; however, their clinical efficacy is frequently constrained by primary or acquired resistance. Harnessing innate immune signaling to increase lymphocyte infiltration into tumors has been recognized a promising approach to augment the anti-cancer immune response to ICBs. Disulfiram (DSF), an FDA-approved drug for chronic alcoholism, has shown potent anti-tumor effect, particularly when used in combination with copper (Cu). Here, we demonstrated a combination treatment of DSF and Cu (DSF/Cu) robustly activated cancer cell-intrinsic cGAS-STING-dependent innate immune signaling pathway. Further studies revealed that DSF/Cu caused mitochondrial and nuclear DNA damage and the release of cytosolic dsDNA by inducing excessive reactive oxygen species (ROS) generation, thereby triggering innate immunity and enhancing anti-tumor effects. Moreover, DSF/Cu significantly increased the intratumoral infiltration of CD8^+^ cytotoxic lymphocytes and natural killer (NK) cells, and potentiated the therapeutic efficacy of PD-1 checkpoint blockade in murine tumor models. Overall, our findings provide a rationale underlying the anti-cancer and immunomodulatory function of DSF/Cu and highlight the potential of repurposing DSF to improve responses to ICBs in cancer patients.

## Introduction

Immunotherapy has emerged as first-line approach in the treatment of various advanced malignancies in recent years. Immune checkpoint blockades (ICBs), represented by monoclonal antibodies to block the PD-1/PD-L1 pathway, exhibit remarkable anti-cancer effect by activating T-cell immune responses within the tumor microenvironment (TME) 1, 2. However, only a fraction of cancer patients benefit from this therapy, mainly due to primary or acquired resistance following ICBs treatment 3, 4. Several mechanisms within the TME have been implicated in the resistance to anti-PD therapy, such as loss of neoantigens and low expression of PD-L1 5. Among these mechanisms, the absence of tumor-infiltrating lymphocytes (TILs) in the TME plays a fundamental role in impairing the immune response. Clinical data show that inflamed or "hot" tumors, which exhibit higher levels of immune cell infiltration, generally respond more effectively to ICBs therapy 6. Thus, enhancing T cell infiltration into tumors is a promising way to transform non-responsive "cold" tumors into responsive "hot" tumors, potentially improving clinical outcomes for anti-PD therapy 7.

The cyclic GMP-AMP synthase (cGAS)-stimulator of interferon genes (STING) pathway is a pivotal component of the innate immune response, which is crucial for detecting cytosolic DNA and mediating protective host defense against infections 8. When cytosolic DNA from pathogens or damaged cells is recognized by cGAS, the enzyme catalyzes the synthesis of cyclic GMP-AMP (cGAMP). Acting as a second messenger, cGAMP then binds to and activates the STING protein, leading to the activation of tank-binding kinase 1 (TBK1)/ transcription of interferon regulatory factor 3 (IRF3) signaling cascades via phosphorylation, ultimately driving the production of type I interferons (IFNα/β) and the downstream pro-inflammatory cytokines 9. Recent studies have revealed that cancer cell-intrinsic cGAS-STING pathway plays crucial roles in shaping an inflamed TME 10. Genomic or mitochondrial DNA damage, induced by radiation and chemotherapy, as well as the activation of endogenous retroviral elements, can lead to the generation of aberrant cytosolic double-stranded DNA (dsDNA). This aberrant dsDNA activates the cGAS-STING pathway in cancer cells and promotes production of IFNβ and pro-inflammatory chemokines, which contribute to the recruitment of dendritic cells and immune effector cells into tumor, thereby eliciting an anti-tumor immune response 11. Given its critical role in enhancing anti-tumor immunity, considerable research efforts are focused on developing therapeutic agents that activate the cGAS-STING pathway, such as the direct STING agonists, PARP inhibitors, and paclitaxel, which showed great effect in sensitizing cancer immunotherapy 12-14.

Disulfiram (DSF, also known as tetraethylthiuram disulfide or Antabuse), a drug used for the management of chronic alcoholism, has been widely applied in clinical practice for decades with proven pharmacokinetics and established safety 15. DSF blocks the oxidation of alcohol through its irreversible inactivation of aldehyde dehydrogenase 16. Beyond its primary indication for alcohol dependence, DSF has garnered attention for its potential anticancer effects, initially through a serendipitous clinical observation that a female alcoholism patient with breast cancer experienced complete resolution of metastases following a decade of DSF administration without receiving any additional cancer therapies 17. Subsequent preclinical studies have supported the anti-cancer role of DSF 18-20. Although the precise mechanisms remain to be fully elucidated, recent studies have suggested that DSF exerts its anti-cancer function by inducing reactive oxygen species (ROS)-dependent oxidative stress and apoptosis 21, inhibiting proteasome activity 22, and modulating tumor immune microenvironment 23. Furthermore, DSF has been shown to chelate bivalent metals, with particular affinity for copper (Cu), which greatly enhanced its anti-cancer activity 24-26. However, the role of DSF or DSF/Cu combination in regulating cancer innate immunity and the precise mechanisms involved remain largely unexplored.

In this study, we identified a role of combined treatment of DSF and Cu (II) potently activating cGAS-STING-dependent innate immune signaling pathway in cancer cells by generating excessive ROS to induce DNA damage and cytosolic dsDNA releases. *In vivo* administration of DSF/Cu in murine tumor models significantly increased the abundance of tumor-infiltrating lymphocytes and potentiated anti-tumor response to PD-1 checkpoint blockade. Our findings highlight an unexpected potential of DSF/Cu in triggering cancer innate immunity and suggest a promising therapeutic application of DSF/Cu on enhancing the effectiveness of ICBs in cancer treatment.

## Materials and Methods

### Reagents and antibodies

Disulfiram (#HY-B0240, MCE), Copper (II) chloride dihydrate (#C3279, Sigma-Aldrich), N-acetylcysteine (#HY-B0215, MCE), ISD (#tlrl-isdn, InvivoGen), diABZI (#S8796, Selleck), DMSO (#D8418, Sigma-Aldrich) were purchased from the indicated manufacturers. The information of antibodies used in this study was listed in Supplementary Table S1.

### Cell lines and cell culture

LLC, B16-F10, Hepa1-6, MDA-MB-231, and HepG2 cells were cultured in DMEM (#12800017, Thermo Fisher Scientific) supplemented with 10% (v/v) fetal bovine serum (FBS) (#900-108, GeminiBio), and 1% (v/v) penicillin-streptomycin (P/S) (100 IU/mL and 100 μg/mL respectively, #15140122, Thermo Fisher Scientific). CT26. WT, 4T1, and H460 cells were cultured in RPMI-1640 (#31800022, Thermo Fisher Scientific) supplemented with 10% (v/v) FBS and 1% (v/v) P/S. LOVO cells were cultured in F-12K (#21127022, Thermo Fisher Scientific) supplemented with 10% (v/v) FBS, 1% (v/v) Glutamax (#35050061, Thermo Fisher Scientific) and 1% (v/v) P/S. All cells were cultured at 37 °C and 5% CO_2_. The cell lines used were either obtained from China Center for Type Culture Collection (CCTCC) or Wuhan Pricella Biotechnology Co., Ltd.

### RNA extraction and quantitative reverse transcription PCR (qRT-PCR) assay

For total RNA extraction, cells were seeded in 12-well plates and then treated with the indicated drugs for 24 h before collection. Reagents, buffers, and containers of RNase-free grade were utilized throughout all RNA procedures. Total RNA was isolated using the RNAiso Plus (#9108, Takara) according to the manufacturer's instructions. For qRT-PCR assay, 1 μg of purified RNA was reverse-transcribed into cDNA with PrimeScript™ RT reagent Kit (#RR047A, Takara) and quantitative real-time PCR was performed using Star Lighter SYBR^®^ Green qPCR Mix (#FS-Q1001, Beijing Foreverstar Biotech Co., Ltd) and run on Bio-Rad CFX96 Touch real-time PCR detection system according to the manufacturer's instructions. Relative value of gene-specific mRNA expression was calculated by the 2^(-ΔΔCt) method. All PCR amplification was normalized to *β-actin*. The primers used were listed in Supplementary Table S2.

### ELISA

Cells were seeded in 12-well culture plates and treated with 0.5 μM DSF/Cu for 12 h followed by stimulated with 1 μg/mL ISD for another 12 h. The concentration of IFNβ and CCL5 in collected culture supernatants was measured using LEGEND MAX™ Mouse IFN-β ELISA Kit (#439407, BioLegend) and Mouse RANTES ELISA Kit (#E-EL-M0009, Elabscience Biotechnology Co., Ltd) according to the manufacturer's instructions. The xenograft tumors were isolated and homogenized using a freezing tissue grinder. The homogenates were then centrifuged and supernatants were collected for ELISA. The levels of IFNβ and CCL5 were detected using the same ELISA kits as above. The levels of IFNγ, Granzyme B, and TNFα were measured using Mouse ELISA Kit (#F2182-A, #F2339-A, #F2132-A, Fankew, Shanghai Kexing Trading Co., Ltd, China) according to the manufacturer's instructions.

### Western blotting

Cells were lysed in the culture plates with cell lysis buffer (#WB3100, NCM Biotech Co., Ltd, China) supplemented with protease and phosphatase inhibitors on ice for 30 min. Subsequently, the cell lysates were collected and centrifuged at 12,000 g for 15 mins at 4 °C. The supernatants were tested using BCA Protein Assay Kit (#P0012S, Beyotime Biotechnology) for protein concentration quantification and then boiled with SDS loading buffer at 95 °C for 10 min. Equal amounts of protein were run on 10% SDS-PAGE gels, transferred onto a PVDF membrane, blocked with 5% BSA, and incubated overnight at 4 °C with the specific primary antibodies listed in Supplementary Table S1. Membranes were washed in TBST, incubated with HRP-conjugated secondary antibodies for 90 min at room temperature, and developed using ECL regents followed by visualized using the Tanon 5200 chemical luminescence imaging system.

### Immunofluorescence (IF) and immunohistochemical (IHC) staining

For IF staining of cultured cells, cells were grown on coverslips in 24-well plates and then treated with the indicated drugs for 24 h. After washing twice with PBS, cells were fixed with 4% paraformaldehyde for 15 min at room temperature followed by permeabilization with 0.3% Triton X-100 in PBS for 10 min. Next, cells were incubated in goat serum blocking solution for 30 min and then incubated overnight at 4 °C with the specific primary antibodies. Cells were washed 3 times with PBS and incubated for 1.5 h at room temperature with Alexa Fluor-labeled secondary antibodies. After washing 3 times with PBS, cells were incubated with DAPI for 5 min at room temperature to stain nuclei. Coverslips were mounted with anti-fade mounting medium (#P0126, Beyotime Biotechnology) and examined by confocal microscopy (Leica TCS SP5). For IF staining of tissue sections, the tumors were resected and fixed in 4% paraformaldehyde, embedded in paraffin and sectioned at a thickness of 4 mm using Leica rotary microtome (Leica RM2235). Tumor sections were performed following the procedures including dewaxing, hydration, antigen retrieval, blocking, and incubating with the primary antibodies against CD8 and CD3 overnight at 4 °C. The remaining procedures were performed the same as IF staining of cultured cells. For IHC assays of tissue sections, the tumor sections were prepared the same as above and Ki-67 staining was performed using a universal IHC kits (PV-9000, ZSGB-Bio Co., Ltd, China) according to the manufacturer's instructions. The IHC images were captured by an Olympus DP71172 microscope. ImageJ software was used for automated image analysis.

### Transfection of short interfering RNAs (siRNAs)

siRNA oligonucleotides targeting human CGAS (si-CGAS #1 and si-CGAS #2) and human STING1 (si-STING1 #1 and si-STING1 #2) as well as control siRNA (si-NC) were purchased from Sangon Biotech (Shanghai, China). Their sequences were presented in Supplementary Table S3. Cells were seeded in 12-well culture plates one day before the transfection. The next day, the specific siRNAs were transfected into cells at a final concentration of 50nM using X-treme GENE siRNA Transfection Reagent (#4476093001, Roche) according to the manufacturer's instructions. At 48 h after siRNA transfection, cells were treated with DSF/Cu for another 24h and then harvested for analysis.

### ROS detection

Cells were seeded in 6-well culture plates and treated with the indicated concentration of DSF, CuCl_2_, DSF/Cu, or in combination with 2 mM NAC for 24 h. The cells were then incubated with 5 μM of CellROX® Oxidative Stress Reagents (#C10422, Thermo Fisher Scientific) at 37 °C for 30 min. For some experiments, cells were incubated with 50µM DCFH-DA (#HY-D0940, MCE) at 37 °C for 30 min to detect ROS production. After digestion with trypsin and washing, cells were resuspended in PBS and analyzed using flow cytometry (Beckman CytoFLEX) for quantifications of cellular ROS levels. Data analysis was conducted with the FlowJo software.

### Mitochondrial morphology and membrane potential detection assay

For mitochondrial morphology imaging, cells were seeded in confocal dishes and treated with indicated drugs for 24 h. The growth medium was removed from the dish and cells were incubated with 200 nM Mito-Tracker Deep Red FM (#C1032, Beyotime Biotechnology) dissolved in prewarmed serum-free medium at 37 °C for 20 min in the dark. After the staining was complete, the medium was replaced with fresh prewarmed complete medium and the cells were examined by confocal microscopy (Leica TCS SP5). For mitochondrial membrane potential detection assay, cells were grown in culture plates and treated the same as above. Mitochondrial membrane potential assay kit with JC-1 (#C2006, Beyotime Biotechnology) was used for measurement. Briefly, cells were incubated with JC-1 probe in staining buffer at 37 °C for 20 min in the dark, followed by washing twice with washing buffer. Fresh prewarmed complete medium was then added into the plates and the images were captured by fluorescence microscopy (Nikon Inverted TE300). ImageJ software was used for automated image analysis.

### Cytosolic dsDNA staining

Cells were grown on coverslips in 24-well plates and then treated with the indicated drugs for 24 h. After the treatment, cells were incubated with culture medium containing PicoGreen dsDNA Reagent (400-fold dilution, #P11495, Thermo Fisher Scientific) at 37 °C for 1 h in the dark. Cells were washed and fixed with 4% paraformaldehyde followed by DAPI counterstaining. Coverslips were then mounted with anti-fade mounting medium and examined by confocal microscopy (Leica TCS SP5).

### In vitro T cell killing assay

This assay was conducted as described previously 27. Briefly, 1.25 μg/mL anti-CD3 (#BE0001, BioXcell) was pre-coated to culture plates and incubated at 4 °C overnight. Lymphocytes were obtained from C57BL/6 mouse spleen, resuspended with RPMI-1640 complete medium containing 2 μg/mL of anti-CD28 (#C379, Leinco technologies) and 1000 U/mL IL-2 (#P5907, Beyotime Biotechnology), and added into anti-CD3-coated culture plate for a 72-h culture to induce the activation of T cells. Meanwhile, B16-F10 and LLC cells (~5 × 10^4^) were seeded in 24-well plates. After 24 h, cells were treated with the indicated concentration of DSF/Cu and incubated with activated T cells at the indicated T cells/tumor cells ratio. After 48 h of coculture, the medium and T cells were removed. The surviving tumor cells were carefully washed with PBS for 3 times, fixed with methanol, and stained with crystal violet solution.

### Animal studies

C57BL/6 mice were purchased from Jiangsu Huachuang sino Pharmaceutical Technology Co., LTD. All mice were maintained under specific pathogen-free (SPF) condition. All animal experimental procedures were evaluated and approved by the Animal Ethics Committee of Xi'an Jiaotong University, China (No. XJTUAE2024-2427).

For the LLC tumor model, 6 × 10^5^ LLC cells were suspended in 100 μL PBS and subcutaneously injected into the right flank of 6-week-old female C57BL/6 mice. Six days after tumor inoculation, mice were randomly grouped and treated with DSF/Cu (50 mg/kg DSF, dissolved in DMSO and diluted in corn oil; 0.15 mg/kg CuCl_2_, dissolved in water; gavage daily) or anti-PD-1 antibody (100 μg per mouse, diluted in PBS, i.p. injection on days 7, 10, and 13 post-inoculation). The same dose of corn oil or isotype control antibody were given synchronously. Tumor sizes were measured every two days with a vernier caliper and tumor volumes were calculated using the following formula: width^2^ × length × 0.5. Mice were sacrificed when tumors reached 15 mm in length and tumors were isolated for further analysis and experiments. For the lung metastasis model, 5 × 10^5^ B16-F10 cells were suspended in 100 μL PBS and injected into the tail vein of 6-week-old male C57BL/6 mice. Six days after tumor inoculation, mice were randomly grouped and treated with DSF/Cu or anti-PD-1 antibody in accordance with above (anti-PD-1 antibody treated on days 9, 12, 15, and 18 post-inoculation). Mice were monitored and weighed every 2 days. After 2-week treatment, mice were sacrificed and tumor-bearing lungs were isolated for the following experiments. Mouse survival was assessed based on endpoints of weight loss exceeding 20% or a decline in behavioral conditions.

To deplete CD8^+^ T and NK cells, mice were injected intraperitoneally with 200μg of anti-mouse CD8α (clone 2.43, Selleck), 200μg of anti-mouse NK1.1 (clone PK136, Selleck), or same dose of isotype control antibody two days prior to tumor inoculation. The depletion was then maintained with repeat doses every 4 days until the end of the treatment. Xenograft tumor models were established by subcutaneously injecting 6 × 10⁵ LLC cells into the right flank of 6-week-old female C57BL/6 mice. Six days post-inoculation, mice were treated orally with DSF/Cu daily at the previously described dose until day 16. Anti-PD-1 antibody was administrated with the same dose and route as previously described on days 7, 9, 12, and 15 post-inoculation. Mice were monitored and weighed every 2 days. At the end of treatment, one mouse from each group was sacrificed for depletion efficiency assessment, and the remaining mice were followed for survival analysis until tumor volume reached 1500 mm³, weight loss exceeded 20%, or signs of severe behavioral decline were observed.

### Flow cytometry analysis of immune cells

For flow cytometry analysis of tumor-infiltrating leukocytes, tumors were excised and dissociated into single-cell suspension by enzymatic digestion followed by filtered through a 70 mm filter. Cells were washed and red cells were removed with lysis buffer. After blocking Fc receptors through incubation with anti-mouse CD16/32 antibody, cells were stained with fluorescence-labeled anti-mouse monoclonal antibodies (listed in Supplementary Table S1) to detect specific superficial markers of CD8^+^T cells and NK cells. 7-AAD was used for distinguishing live cells from the total cell population. Samples were analyzed by flow cytometry, and data analysis was conducted with the FlowJo software. For immune cell depletion analysis, spleen and peripheral blood samples were collected from mice and processed for CD8^+^T and NK cells staining. CD8^+^T cells were detected using anti-CD8α (clone 53-6.7) and NK cells with anti-CD49b (clone DX5), ensuring no competition with depletion antibodies for epitopes. The representative flow cemetery gating strategies for CD8^+^T cells and NK cells were shown in Supplementary Figure S9.

### Biosafety evaluation

The toxicity of DSF/Cu and anti-PD-1 treatment was evaluated through measurements of body weight, hepatic and renal function, and microscopic structures of viscera. Serum samples from the indicated groups were collected and examined for the levels of glutamic pyruvic transaminase (GPT/ALT), glutamic oxaloacetic transaminase (GOT/AST), creatinine (CRE), and urea nitrogen (BUN) with specific ELISA kits (#C009-2-1, #C010-2-1, #C011-2-1, #C013-2-1, Nanjingjiancheng Bioengineering Institute, China) according to the manufacturer's instructions. The microscopic structures of mice organs were observed by hematoxylin-eosin (H&E) staining of paraffin-embedded tissues under an Olympus DP71172 microscope.

### Statistical analysis

Statistical analyses were performed using GraphPad Prism software (v8.0). Statistical analysis details for the different experiments are reported in figure legends or the methods section. p < 0.05 was recognized as a statistically significant difference.

## Results

### Combined treatment of DSF and Cu (II) robustly activates cGAS-STING-dependent innate immunity in cancer cells

**Figure 1:**
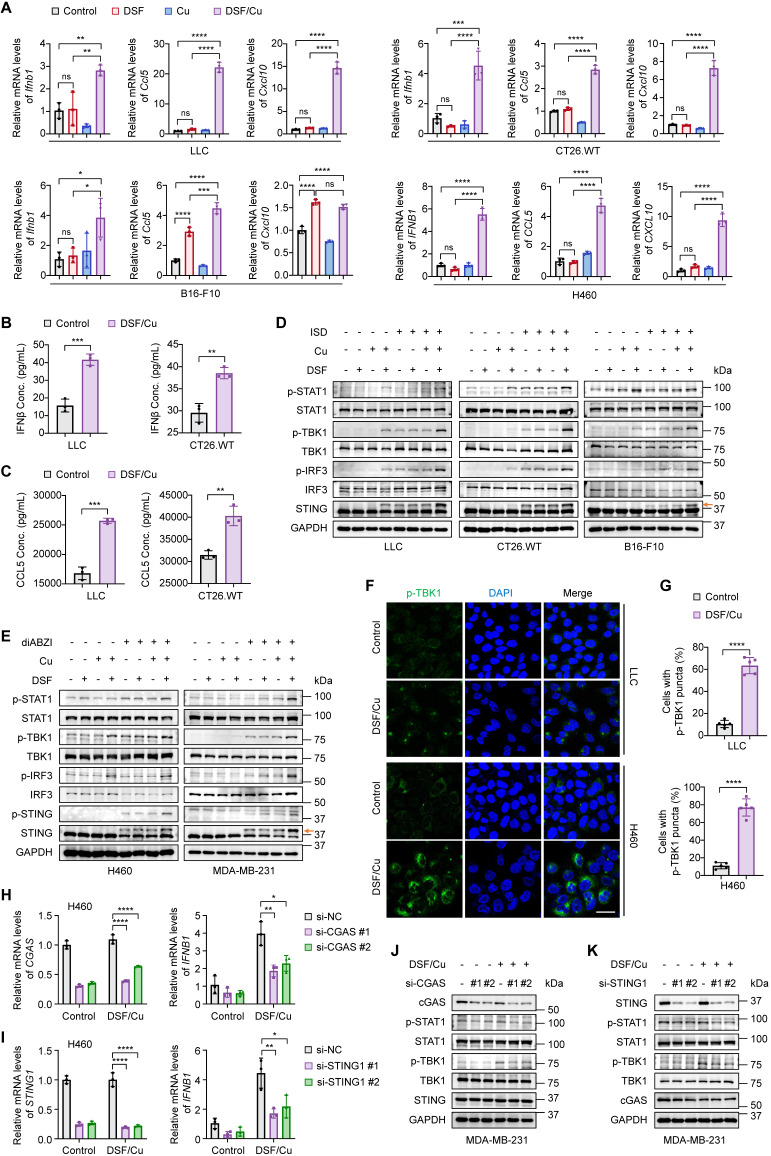
** Activation of cGAS-STING-dependent cancer cell-intrinsic innate immunity by combined treatment of DSF and Cu.** Several mouse or human cancer cell lines were treated with DSF (1 μM for H460 and 0.5 μM for others) and CuCl_2_ (1 μM for H460 and 0.5 μM for others), individually or in combination (at 1:1 molar ratio), for 24 h. Control groups were synchronously treated with the same volume of DMSO or water. (**A**) qRT-PCR was performed to detect mRNA levels of IFNB1, CCL5, and CXCL10 in indicated cells. (**B-C**) ELISA was used to measure the production of mouse IFNβ (B) and CCL5 (C) in the culture supernatants of the indicated cells upon DSF/Cu treatment. (**D-E**) Immunoblotting analysis of the indicated proteins in mouse (D) and human (E) cancer cells treated with DSF and/or CuCl_2_, followed by 1 μg/mL ISD stimulation for 4 h or 5 μM diABZI stimulation for 2 h. The orange arrow points to the band of phosphorylated STING. (**F**) Representative fluorescence images of DAPI (blue) and p-TBK1 (green) staining in LLC and H460 cells. Scale bar, 30 μm. (**G**) Quantification of the percentage of cells with one or more p-TBK1 puncta. H460 and MDA-MB-231 cells were transfected with siRNAs targeting CGAS or STING for 48 h and then treated with 1 μM DSF/Cu for another 24 h. (**H-I**) qRT-PCR was performed to detect mRNA levels of CGAS, STING1, and IFNB1 in H460 cells treated as indicated. (**J-K**) Immunoblotting analysis of the indicated proteins in MDA-MB-231 cells. All data are shown as mean ± SD of independent biological replicates, and p values were determined by unpaired Student's t test (for two groups data) or one-way ANOVA followed by Tukey's multiple comparisons test (for four groups data). ****p < 0.0001, ***p < 0.001, **p < 0.01, *p < 0.05; ns, not significant.

To determine the effect of disulfiram (DSF) on cell-intrinsic innate immunity, we treated a panel of murine and human cell lines representing multiple cancer types with a series of concentrations of DSF and Copper (II) chloride (Cu), individually or in combination. DSF or Cu treatment alone had minimal effect on activating type I interferon *IFNB1* expression, especially at concentrations lower than 10µM (Fig. 1A; Supplementary Figures S1A-B and S2A). However, combined treatment of DSF and Cu in equivalent dose greatly boosted *IFNB1* expression at extremely low concentrations (Fig. 1A and Supplementary Figure S2A). We also treated cancer cells with various Cu concentrations combined with a fixed dose of DSF. qRT-PCR results showed that DSF induced *IFNB1* expression in a Cu-dependent manner (Supplementary Figure S1C), further confirming the essential role of Cu in DSF-mediated activation of cancer cell-intrinsic innate immunity.

Tumor-secreted IFNβ binds to the IFNAR1/2 receptors on antigen-presenting cells and tumor cells, inducing the release of proinflammatory chemokines, such as CCL5 and CXCL10 28. These chemokines, through binding to CCR5 and CXCR3 receptors on T and NK cells, promote their migration from tumor-draining lymph nodes to the TME where they can recognize tumor antigens and become activated to eliminate tumor cells 28, 29. The expression of CCL5 and CXCL10 has been strongly associated with an inflamed TME and improved patient survival 29, 30. We then assessed their expression upon DSF and Cu treatment. Combined treatment of DSF and Cu, but not either agent alone, significantly increased *CCL5* and* CXCL10* mRNA levels at low concentrations (Fig. 1A and Supplementary Figure S2A). Consistently, the concentrations of secreted IFNβ and CCL5 in cell culture supernatants were significantly elevated upon DSF/Cu treatment (Fig. 1B-C).

The cGAS-STING-dependent innate immune signaling pathway is marked by a phosphorylation cascade involving TBK1, IRF3, and STING, as well as the subsequent activation of the IFNβ-mediated JAK/STAT pathway, evidenced by increased phosphorylation of STAT1 8, 31. Due to the extremely low basal levels of these phosphorylated proteins in cancer cells, we utilized two specific stimulators to facilitate their detection. Western blotting results demonstrated greatly increased phosphorylation levels of STING, TBK1, IRF3, and STAT1 in response to DSF/Cu treatment when stimulated either by a dsDNA analogue ISD (Fig. 1D and Supplementary Figure S2B) or by direct activation of STING with diABZI (Fig. 1E and Supplementary Figure S2C) 32. This was further supported by immunofluorescence experiments showing that phosphorylated TBK1 was strongly enhanced in amounts and accumulated to form more puncta after DSF/Cu treatment (Fig. 1F-G and Supplementary Figures S2D-E). Additionally, we found that DSF/Cu significantly activated cGAS-STING pathway and promoted IFNβ production in both a dose- and time-dependent manner (Supplementary Figures S3A-F).

The production of IFNβ is not only mediated by cGAS-STING pathway but also by the dsRNA sensing pathway^33^. To establish the essential roles of cGAS and STING in DSF/Cu-induced IFNβ production, we genetically silenced these molecules using siRNAs. The results showed that the elevated expression of IFNB1 induced by DSF/Cu treatment was largely abolished upon cGAS or STING depletion. (Fig. 1H-I). Consistently, the knockdown of either cGAS or STING significantly reduced the phosphorylation levels of TBK1 and STAT1 in response to DSF/Cu. (Fig. 1J-K). Taken together, our data demonstrate that combined treatment of DSF and Cu robustly triggers cGAS-STING-mediated innate immunity in cancer cells.

### DSF/Cu triggers nuclear and mitochondrial DNA damage and cytoplasmic release of dsDNA by inducing reactive oxygen species (ROS) generation

Considering the consistency and reproducibility of our results across multiple cancer cell lines, we speculated that DSF/Cu might activate cancer cell-intrinsic innate immunity through a shared mechanism. Oxidative stress induced by excessive ROS generation is considered one of the primary mechanisms through which DSF exerts its anti-tumor effect 34. ROS are important byproducts of cellular metabolism, and their levels are tightly regulated under physiological conditions. Moderate increase of ROS levels can promote tumor progression, whereas severe or persistent ROS production leads to irreversible DNA damage and cell death 35. In addition, cellular ROS accumulation inevitably causes intrinsic damage of both nuclear and mitochondrial DNA, resulting in the aberrant presence of cytosolic double-stranded DNA (dsDNA) fragments, which serve as strong agonist of cGAS-STING-dependent innate immune signaling pathway 36, 37. Moreover, previous studies have shown that DSF/Cu enhances temozolomide- or radiation-induced DNA damage in glioblastoma cells 38. Based on these facts, we hypothesized that DSF/Cu might activate cell-intrinsic innate immunity by inducing excessive ROS generation and DNA damage.

**Figure 2:**
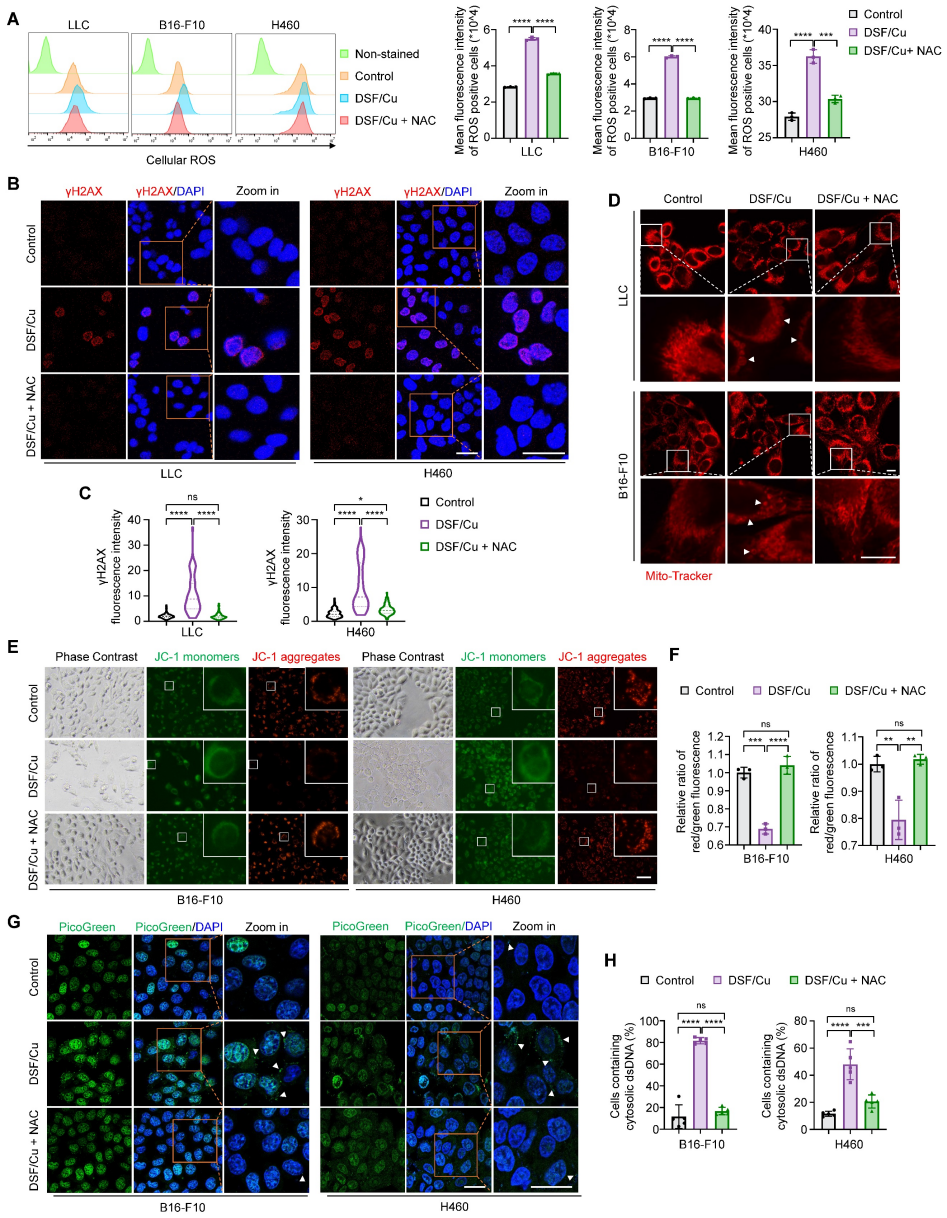
** DSF/Cu induces ROS generation to trigger nuclear and mitochondrial DNA damage and cytoplasmic release of dsDNA.** The indicated cells were treated with DSF/Cu (1 μM for H460 and 0.5 μM for others, at 1:1 molar ratio) alone or in combination with 2 mM NAC for 24 h. (**A**) Representative flow cytometry results showed cellular ROS levels of cells treated as indicated. Right panel indicated the quantitative analysis of ROS levels based on the mean intensity of the CellROX® Deep Red fluorescence on flow cytometer. (**B**) Representative images of γH2AX foci (red) and DAPI nuclear staining (blue) in LLC and H460 cells treated as indicated. Scale bars, 30 μm. (**C**) The fluorescence intensity of more than 50 cells per group was quantified. (**D**) Representative images of mitochondrial morphology of cells treated as indicated. Cells was labeled with a mitochondrial-specific fluorescence probe, Mito-Tracker Deep Red FM. White triangles indicate fragmented or swollen mitochondria. Scale bars, 10 μm. (**E**) Representative images of mitochondrial membrane potential changes tested by JC-1 fluorescence probe in cells treated as indicated. Green fluorescence showed JC-1 as monomers when mitochondrial membrane potential was in a low level, while red fluorescence showed JC-1 aggregating in mitochondrial matrix when mitochondrial membrane potential was in a high level. (**F**) Quantitative analysis of the relative ratios of JC-1 red/green fluorescence in the indicated groups. Scale bar, 100 μm. (**G**) Representative images of PicoGreen staining (green) and DAPI nuclear staining (blue) in B16-F10 and H460 cells treated as indicated. Scale bars, 30 μm. (**H**) Quantification of the percentage of cells containing cytosolic dsDNA foci. All data are presented as mean ± SD of independent biological replicates, and p values were determined by one-way ANOVA followed by Tukey's multiple comparisons test. ****p < 0.0001, ***p < 0.001, **p < 0.01; ns, not significant.

As expected, combined treatment of DSF and Cu significantly increased cellular ROS levels (Fig. 2A and Supplementary Figure S4A), in line with previous studies 39, 40. N-acetylcysteine (NAC), a glutathione precursor with antioxidant properties, effectively cleared the excessive ROS induced by DSF/Cu (Fig. 2A and Supplementary Figure S4A). In addition, we found that DSF or Cu alone also contributed to ROS production, while DSF/Cu combination treatment further enhanced cellular ROS level (Supplementary Figures S5A-B). Notably, treatment of LLC cells with 30 μM DSF led to high ROS levels (Supplementary Figure S5B), in agreement with our previous findings that 30 μM DSF significantly induced *IFNB1* expression (Supplementary Figure S1A). This suggests that excessive ROS generation is required for DSF/Cu-induced innate immunity activation.

**Figure 3:**
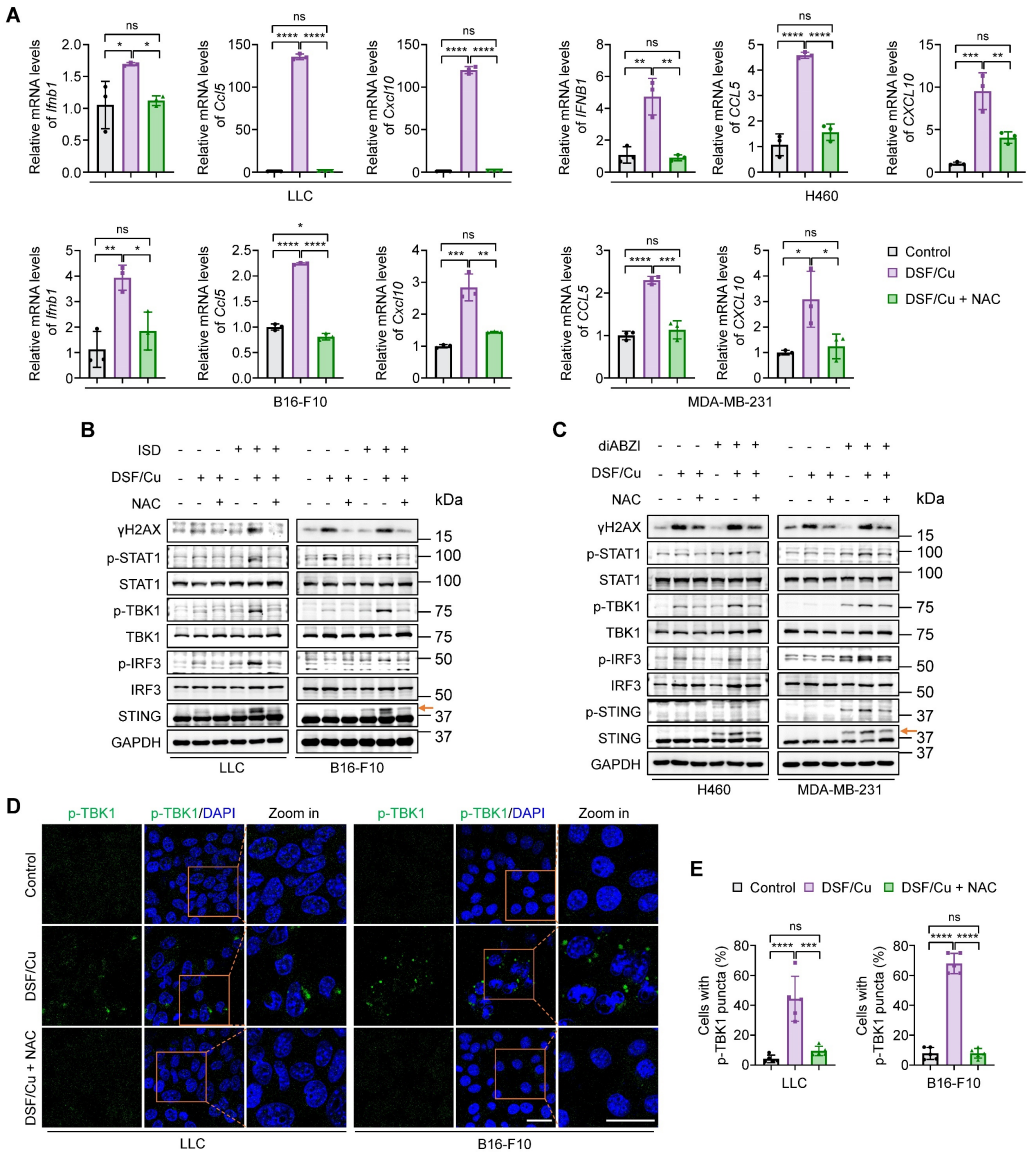
**Blockage of DSF/Cu-induced innate immunity activation by ROS clearance.** Cells were treated with DSF/Cu (1 μM for H460 and 0.5 μM for others, at 1:1 molar ratio) alone or in combination with 2 mM NAC for 24 h. (**A**) qRT-PCR was performed to detect mRNA levels of IFNB1, CCL5, and CXCL10 in indicated cells. (**B-C**) Immunoblotting analysis of the indicated proteins in mouse (B) and human (C) cancer cells treated with DSF/Cu, followed by 1 μg/mL ISD stimulation for 4 h or 5 μM diABZI stimulation for 2 h. The orange arrow points to the band of phosphorylated STING. (**D**) Representative fluorescence images of DAPI and p-TBK1 staining in LLC and B16-F10 cells treated as indicated. Scale bars, 30 μm. (**E**) Quantification of the percentage of cells with one or more p-TBK1 puncta. All data are shown as mean ± SD of independent biological replicates, and p values were determined by one-way ANOVA followed by Tukey's multiple comparisons test. ****p < 0.0001, ***p < 0.001, **p < 0.01, *p < 0.05; ns, not significant.

To demonstrate that DSF/Cu induces DNA damage by generating excessive ROS, we firstly detected the levels of γH2AX, a molecular marker of DNA double-strand breaks. Confocal microscopic images and western blotting results consistently showed that DSF/Cu-treated cells displayed increased levels of γH2AX, while the clearance of ROS by NAC effectively restored this effect (Fig. 2B-C and 3B-C; Supplementary Figures S4B-C), which was consistent with a previous study 38. Severe oxidative stress can lead to mitochondrial network remodeling, characterized by fragmented or swollen morphology and a loss of mitochondrial membrane potential 41. Thus, we employed Mito-Tracker Deep Red probe to label the mitochondria and JC-1 fluorescence probe to measure mitochondrial membrane potential, respectively. The results showed that DSF/Cu induced distinct mitochondrial fragmentation and swollenness, while the clearance of ROS by NAC largely restored the mitochondrial morphology to a tubular and densely packed normal state (Fig. 2D). Moreover, a significant decrease in mitochondrial membrane potential was observed after DSF/Cu treatment, but not upon NAC rescue, as evidenced by a reduced ratio of red JC-1 aggregates to green JC-1 monomers (Fig. 2E-F; Supplementary Figures S4D-E).

To further investigate whether DSF/Cu facilitates damaged nuclear or mitochondrial DNA leakage into the cytoplasm, we therefore assessed the cytosolic dsDNA with PicoGreen staining. The results indicated that DSF/Cu treatment resulted in a significantly higher proportion of cells harbored cytosolic dsDNA foci compared to the control, and this effect could be reversed by NAC treatment (Fig. 2G-H; Supplementary Figures S4F-G). Collectively, our results indicate that DSF/Cu induces oxidation stress, DNA damage and cytoplasmic release of dsDNA by increasing cellular ROS levels.

### ROS clearance blocks DSF/Cu-induced activation of innate immunity

Given the essential role of ROS generation in DSF/Cu-induced cytosolic dsDNA release, we next examined the effect of ROS clearance on cell-intrinsic innate immune signaling pathway. DSF/Cu significantly enhanced the levels of IFNβ and its downstream proinflammatory chemokines, CCL5 and CXCL10, in comparison with the control, while this effect was successfully reversed by ROS clearance with NAC (Fig. 3A). The elevated phosphorylation levels of key mediators involved in cGAS-STING-dependent innate immune signaling pathway in response to DSF/Cu, including p-STING, p-TBK1, p-IRF3, and p-STAT1, were largely reverted to the levels comparable to the control group upon NAC treatment when stimulated either by ISD or diABZI (Fig. 3B-C). Consistently, immunofluorescence results showed that ROS clearance significantly decreased the intensity of phosphorylated TBK1 and the proportion of cells with p-TBK1 puncta around the nucleus in response to the treatment of DSF/Cu (Fig. 3D-E).

NAC has been shown to suppress TNF-induced NF-κB activation by inhibiting IκB kinases 42. Given that activated STING can stimulate IKK-IκBα kinase-mediated NF-κB phosphorylation and nuclear translocation, facilitating IRF3-driven IFNβ expression 9, and that NF-κB activation promotes STING trafficking and activation 43, it is possible that NAC inhibits NF-κB, thereby contributing to the suppression of innate immune activation independently of its ROS-scavenging function. To exclude potential off-target effects, we treated cells with NAC alone and found that 2 mM NAC—the same concentration used in the DSF/Cu plus NAC group—had no obvious inhibitory effect on both NF-κB p65 and STING-TBK1-IRF3 activation compared to the control group, as evidenced by the phosphorylation levels of these proteins (Supplementary Figure S6A). qRT-PCR results consistently indicated that NAC alone did not alter *IFNB1* expression (Supplementary Figure S6B). These findings, taken together, indicate that DSF/Cu induces innate immunity activation in a ROS-dependent manner.

### DSF/Cu suppresses tumor growth and potentiates therapeutic efficacy of anti-PD-1 antibody in murine tumor models

**Figure 4:**
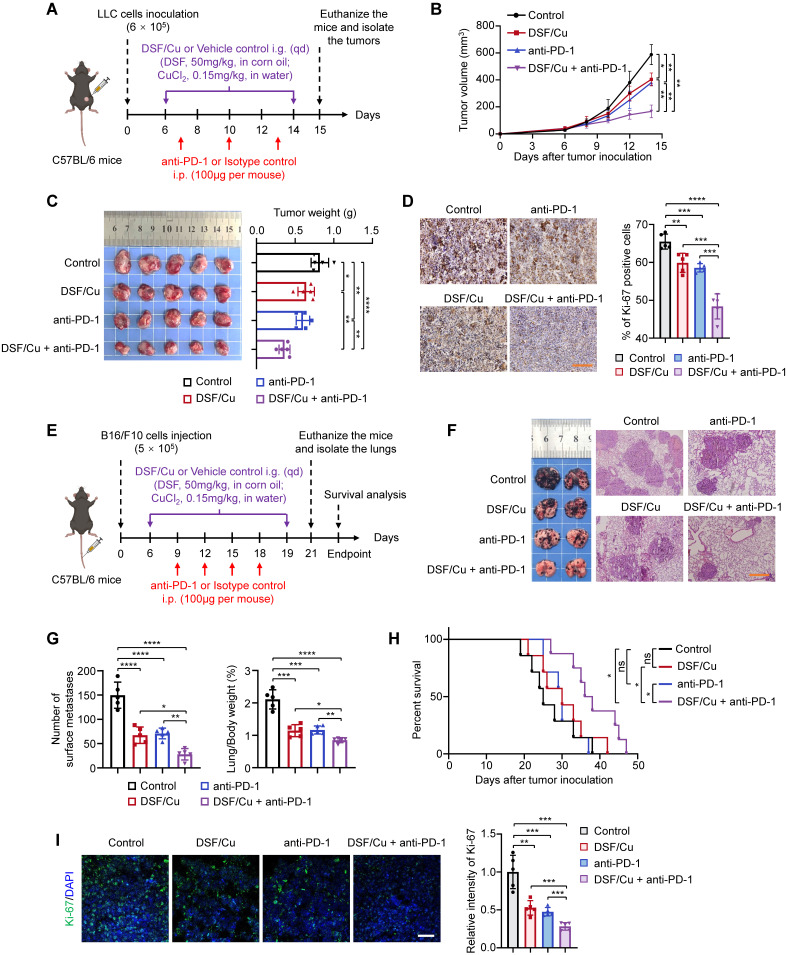
** DSF/Cu suppresses tumor growth and potentiates the efficacy of anti-PD-1 therapy in mice.** (**A**) Schematic diagram of LLC subcutaneous tumor model and treatment process of DSF/Cu and anti-PD-1 antibody. LLC cells (6 × 10^5^) were subcutaneously injected into the right flank of C57BL/6 mice, and mice were then randomly grouped (n =5 per group) and treated with DSF/Cu (DSF 50 mg/kg; CuCl_2_ 0.15 mg/kg) or anti-PD-1 antibody (100μg per mouse) for the indicated times. The same dose of corn oil or isotype antibody were given synchronously as the control. All mice were sacrificed on day 15 and xenograft tumors were isolated for the following studies. i.g., oral gavage; i.p., intraperitoneal injection; qd, quaque die/every day. (**B**) Growth curves of LLC tumors of each group are shown (n = 5). Tumor growth was measured every 2 days and further analyzed by two-way ANOVA followed by Tukey's multiple comparisons test. (**C**) Representative images showing the volumes of LLC tumors (left panel), and quantitative analysis of tumor weights from the indicated groups (right panel). (**D**) Representative IHC images for Ki-67 staining of LLC tumor sections in the indicated groups (left panel), and quantification of the percentage of Ki-67 positive cells (right panel). Scale bar, 100 μm. (**E**) Schematic diagram of B16-F10 lung metastasis model and treatment process of DSF/Cu and anti-PD-1 antibody. B16-F10 cells (5 × 10^5^) were injected into the tail vein of C57BL/6 mice, and mice were then randomly grouped (n =12~13 per group) and treated with DSF/Cu (DSF 50 mg/kg; CuCl_2_ 0.15 mg/kg) or anti-PD-1 antibody (100 μg per mouse) for the indicated times. The same dose of corn oil or isotype antibody were given synchronously as the control. Some of the mice were sacrificed on day 19 and tumor-bearing lungs were isolated for the following studies. i.g., oral gavage; i.p., intraperitoneal injection. qd, quaque die/every day. (**F**) Representative images of melanoma-bearing lungs (left panel) and H&E stained-lung sections (right panel) from the indicated groups (n = 5). Scale bar, 400 μm. (**G**) Quantitative analysis for number of surface metastatic nodules (left panel) and the ratios of lung weight to body weight of mice from the indicated groups (right panel). (**H**) Survival curves of B16-F10 tumor-bearing mice after treatments are shown and were analyzed using the Kaplan-Meier method. p value was calculated by log rank test. (n = 8 for the group of DSF/Cu + anti-PD-1, n = 7 for the other groups). (**I**) Representative IF images for Ki-67 staining (green) of B16-F10 tumor sections in the indicated groups (left panel) and quantification of relative fluorescence intensity of Ki-67 staining (right panel) (n = 5). Scale bar, 50 μm. All data are shown as means ± SD, and p values were calculated by one-way ANOVA followed by Tukey's multiple comparisons test unless otherwise specified. ****p < 0.0001, ***p < 0.001, **p < 0.01, *p < 0.05.

To evaluate the anti-tumor effect of DSF/Cu *in vivo*, we firstly established an LLC subcutaneous tumor-bearing C57BL/6 mouse model and treated the mice with DSF/Cu and anti-mouse PD-1 *in vivo* antibody, individually or in combination (Fig. 4A). DSF/Cu caused a decrease in tumor growth and tumor weight compared with control tumors (Fig. 4B-C), consistent with a previous study 44. A combination treatment of DSF/Cu and anti-PD-1 antibody further retarded the growth of LLC tumors (Fig. 4B) and resulted in a significant reduction in tumor weight (Fig. 4C) compared with DSF/Cu or anti-PD-1 monotherapy. The IHC results also showed a substantial decrease in the percentage of Ki-67-positive cells in the tumor sections of combination therapy group in comparison with other groups (Fig. 4D), further supporting the above conclusions.

Tumor metastasis is a major factor contributing to poor clinical prognosis and patient death 45. Immune checkpoints inhibitors (ICIs) are commonly employed to treat metastatic tumors, but a large proportion of patients with metastatic disease continue to exhibit resistance to ICBs 5. Therefore, we next attempted to evaluate the efficacy of DSF/Cu in treating metastatic tumors and its potential to enhance the sensitivity of ICBs by a lung metastatic B16-F10 melanoma model (Fig. 4E). As expected, DSF/Cu-treated mice had less metastatic nodules in lungs than those in the control group, while combination treatment of DSF/Cu and anti-PD-1 antibody displayed superior efficacy in inhibiting metastatic tumor growth and improving mouse survival compared with DSF/Cu or anti-PD-1 monotherapy (Fig. 4F-H). In addition, the percentages of Ki-67-positive cells were significantly decreased in tumor sections of mice from combination treatment group (Fig. 4I).

**Figure 5:**
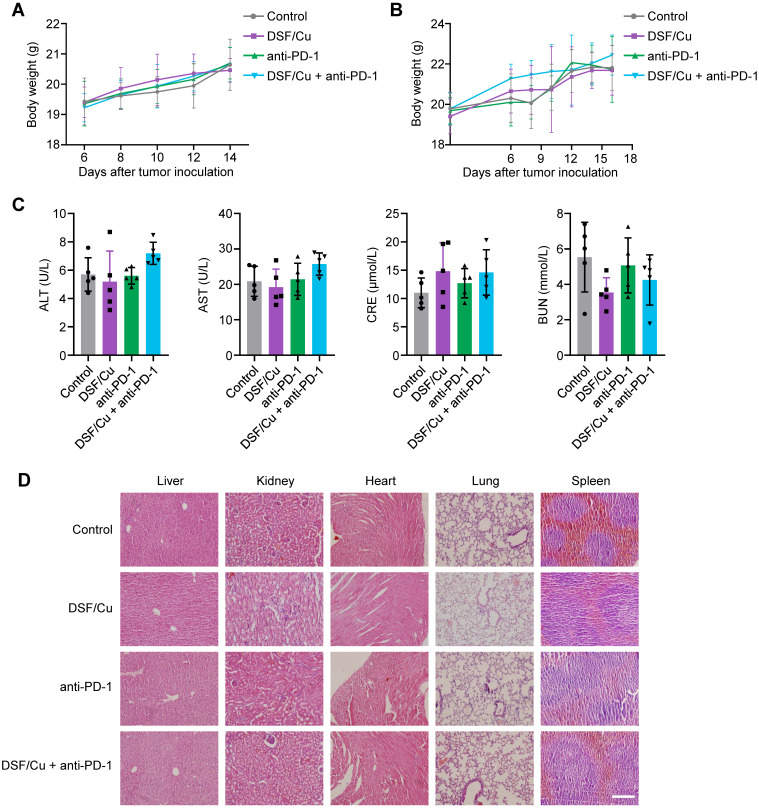
***In vivo* biosafety assessment of DSF/Cu.** (**A-B**) Body weight curves of LLC tumor-bearing mice (A) and B16-F10 tumor-bearing mice (B) from the indicated groups are shown (n = 5). Body weights of mice were measured every 2 days and further analyzed by two-way ANOVA. (**C**) ELISA was used to measure the levels of alanine transaminase (ALT), aspartate aminotransferase (AST), serum creatinine (CRE), and blood urea nitrogen (BUN) in serum of mice with the indicated treatments. Data were analyzed by one-way ANOVA. (**D**) Representative images of H&E-stained organ sections of LLC tumor-bearing mice with the indicated treatments. Scale bar, 200 μm.

As an FDA-approved drug for treating alcoholism, DSF has been applied in clinical practice for decades with great safety. We evaluated the biosafety of DSF/Cu during the course of tumor therapy by monitoring body weight and measuring the function of major organs in mice. The results showed no significant difference in body weight among these four groups in both subcutaneous and metastatic tumor models (Fig. 5A-B). Also, no obvious hepatic and renal toxicity were observed among the indicated treatment groups, as assessed through the levels of alanine transaminase (ALT), aspartate aminotransferase (AST), serum creatinine (CRE), and blood urea nitrogen (BUN) in the serum of mice (Fig. 5C). H&E staining of organ sections consistently indicated that DSF/Cu was well-tolerated and caused no obvious tissue damage (Fig. 5D). These findings suggest that DSF/Cu effectively inhibits tumor progression and improves the efficacy of anti-PD-1 therapy, with a good biosafety profile.

### DSF/Cu enhances anti-tumor immune response

**Figure 6:**
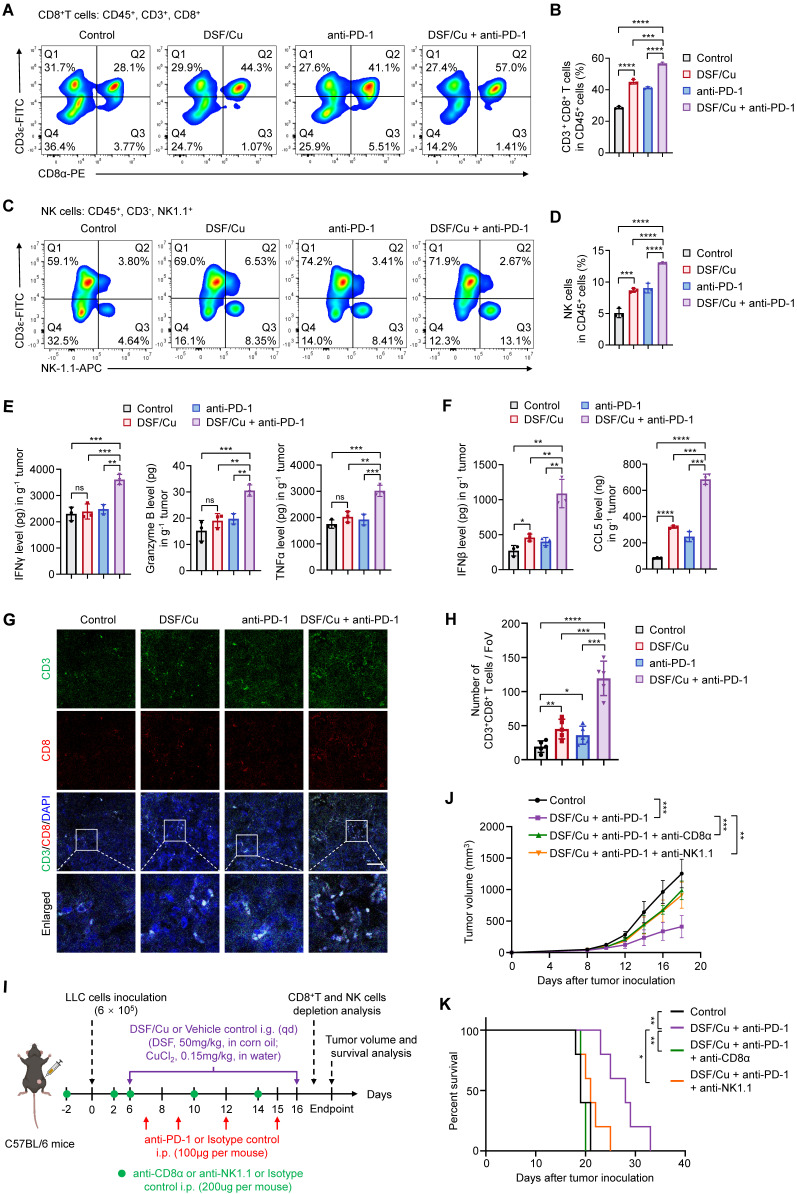
** Combined treatment of DSF/Cu and anti-PD-1 antibody enhances anti-tumor immune response in mice.** (**A-B**) Representative images of flow cytometry analysis (A) quantification (B) of the tumor-infiltrating CD8^+^ T cells in LLC tumors of the indicated groups. (**C-D**) Representative images of flow cytometry analysis (C) quantification (D) of the tumor-infiltrating NK cells in LLC tumors of the indicated groups. (**E**) The production of IFNγ, Granzyme B, and TNFα in LLC tumors of the indicated groups were measured by ELISA. (**F**) ELISA was used to measure the levels of chemokines IFNβ and CCL5 in LLC tumors with the indicated treatments. (**G**) Representative images for IF staining of CD3 (green), CD8 (red), and DAPI (blue) in B16-F10 tumor sections of the indicated groups. Scale bar, 50 μm. (**H**) Quantitative analysis for the numbers of CD3^+^CD8^+^ T cells in the above tumor sections (n = 5 fields of view [FoV]). (**I**) Schematic diagram of immune cell depletion model and treatment process of DSF/Cu, anti-PD-1 antibody, and depletion antibodies. LLC cells (6 × 10^5^) were subcutaneously injected into the right flank of C57BL/6 mice (n =6 per group) and treated with DSF/Cu (DSF 50 mg/kg; CuCl_2_ 0.15 mg/kg), anti-PD-1 antibody (100μg per mouse), anti-CD8α antibody (200μg per mouse), or anti-NK1.1 antibody (200μg per mouse) for the indicated times. The same dose of corn oil or isotype antibody were given synchronously as the control. i.g., oral gavage; i.p., intraperitoneal injection; qd, quaque die/every day. (**J**) Growth curves of LLC tumors of each group are shown (n = 5). Tumor growth was measured every 2 days and further analyzed by two-way ANOVA followed by Tukey's multiple comparisons test. (**K**) Survival curves of LLC tumor-bearing mice after treatments are shown and were analyzed using the Kaplan-Meier method. p value was calculated by log rank test (n = 5). All data are shown as means ± SD, and p values were calculated by one-way ANOVA followed by Tukey's multiple comparisons test unless otherwise specified. ****p < 0.0001, ***p < 0.001, **p < 0.01, *p < 0.05; ns, not significant.

Considering that DSF/Cu robustly activates innate immunity* in vitro,* we next investigated its effect on tumor immune microenvironment of the murine models. Flow cytometry analysis showed that a combination treatment of DSF/Cu and anti-PD-1 antibody increased the intratumoral infiltration of CD8^+^ T and NK cells (Fig. 6A-D), which serve as the primary effector cells in anti-tumor immunity. The release of effector cytokines is one of major pathways mediating tumor killing function of CD8^+^ T and NK cells 46. Thus, we tested the production levels of IFNγ, Granzyme B, and TNFα in LLC tumors by ELISA. The results showed that DSF/Cu had negligible effect on the production of cytokines, while combination treatment of DSF/Cu and anti-PD-1 antibody considerably enhanced intratumoral abundance of these cytokines (Fig. 6E). In addition, the levels of IFNβ and CCL5 were greatly increased in the tumors from combination treatment group, indicating effective activation of innate immunity *in vivo* (Fig. 6F). IF staining of B16-F10 lung metastases sections confirmed enhanced infiltration of CD8^+^ T cells in the tumors with combination treatment of DSF/Cu and anti-PD-1 antibody (Fig. 6G-H). An *in vitro* coculture system of mouse primary T cells and tumor cells was applied to further assess the antitumor immune response of DSF/Cu treatment. Notably, DSF/Cu enhanced T cell-mediated tumor cell killing in a dose-dependent manner (Supplementary Figure S7).

To further establish the contributions of CD8^+^ T and NK cells in DSF/Cu and anti-PD-1-mediated tumor growth inhibition, we depleted these cells in LLC-tumor bearing mice using specific antibodies (Fig. 6I). Flow cytometry confirmed significant depletion of CD8^+^ T and NK cells in the spleens and peripheral blood of treated mice (Supplementary Figure S8). Depletion of both CD8^+^ T and NK cells largely reversed tumor growth (Fig. 6J) and mice survival (Fig. 6K) in response to DSF/Cu and anti-PD-1 combination therapy, suggesting that CD8^+^ T and NK cells are key effectors of the observed antitumor response. Taken together, these results demonstrate that DSF/Cu fuels immune cells infiltration and boosts anti-tumor immune response of anti-PD-1 therapy.

## Discussion

Targeting cGAS-STING-dependent immune signaling pathway has been proved to be an effective strategy to enhance anti-cancer immunity and sensitive tumors to ICBs therapy 47. Cancer cells often exhibit reduced activity of cGAS-STING pathway compared to normal cells, largely due to various negative regulation mechanisms which dampen the activity of the STING protein 11. This has driven the development of specific STING agonists, such as cyclic dinucleotide (CDN) analogues ADU-S100 and MK-1454, as well as the non-CDN small molecule GSK-3745417. Despite their promising results in pre-clinical studies, several obstacles hinder the clinical application of the STING agonists and compromise their therapeutic efficacy, including poor *in vivo* stability, challenges in systemic administration, and species- or structure-specific selectivity. For example, CDN analogs have limited membrane permeability and are prone to hydrolysis, often necessitating intratumoral injection 12. Furthermore, some STING agonists, such as DMXAA, have shown efficacy in mice but failed in human trials due to their inability to bind human STING 48. Additionally, single nucleotide polymorphisms (SNPs) in the STING gene can alter the protein structure, thereby affecting its sensitivity to specific agonists 49. In contrast, both DSF and Cu (II) are stable and can be effectively absorbed and metabolized when administered orally. Our results also demonstrated that DSF/Cu activate the cGAS/STING pathway through ROS production and DNA damage, which are independent of species or structural selectivity. Moreover, DSF/Cu activated innate immunity in both human and mouse cancer cells, providing a broader therapeutic impact. Previous studies have indicated that STING agonists administered alone typically exhibit limited anti-tumor efficacy 50, 51, primarily because the activation of innate immunity alone has a minimal direct effect on cancer cell killing. Their anti-tumor activity largely depends on adaptive immune activation, which is often suppressed within the TME 52. In contrast, DSF/Cu has demonstrated potent anti-cancer effects *in vitro*, reducing tumor growth by 74% in nude mice compared to solvent controls, through mechanisms such as excessive ROS generation and proteasome inhibition. 19, 53. Thus, DSF/Cu may offer a dual benefit: inhibiting cancer cell growth while simultaneously activating innate immunity to enhance T cell-mediated tumor killing, thereby potentially maximize the overall therapeutic efficacy. In summary, DSF/Cu demonstrates significant potential as an immune activator, both in terms of its inherent properties and mode of action, making it promising for clinical translation.

The development of new cancer therapies is a challenging, costly, and time-consuming process. Many drugs, even after reaching the clinical trial stage, still face a high risk of trial termination due to unforeseen toxicity or inadequate therapeutic efficacy. Drug repurposing offers a promising strategy to expedite the clinical trial and approval process while substantially reducing the associated costs 54. DSF, a drug approved by the U.S. Food and Drug Administration (FDA) for the management of alcohol dependence for over seventy years, has demonstrated potential therapeutic applications beyond its original indication, particularly in oncology 18-20. Notably, DSF has shown potent anti-cancer effects, especially when used in combination with Cu 24, 25. In the present study, we confirmed the therapeutic efficacy and biosafety of DSF/Cu in murine tumor models, aligning with the findings of previous studies. DSF/Cu exhibited a significant enhancement in the anti-tumor response to PD-1 checkpoint blockade. Furthermore, ongoing clinical trials are assessing the efficacy and safety of DSF alone and in combination with Cu across various cancer types 55, 56. Thus, DSF/Cu holds considerable promise for cancer therapy and deserves further investigation to determine its potential as a viable treatment option. However, the potential challenges and risks of DSF and Cu in tumor treatment cannot be ignored. Excessive ROS production and Cu accumulation can lead to systemic toxicity and non-specific damage to healthy tissues. Additionally, DSF and Cu may interfere with other biological processes or interact with concurrent treatments, resulting in unexpected adverse effects 56. As aberrant copper accumulation has been shown to contribute to the malignant transformation of cells and promote immune escape by enhancing PD-L1 expression 57, there is a potential risk that the use of copper supplements could promote unforeseen tumorigenesis. Future studies should focus on developing strategies such as precise drug delivery, optimized dosing, and combination therapies to enhance efficacy while minimizing side effects, ensuring the safety and effectiveness of DSF and Cu-based treatments.

A previous study has indicated that DSF combined with chemotherapy activates the cGAS-STING pathway in pancreatic cancer cells by inhibiting PARP1 activity; however, DSF alone showed no effect on enhancing *IFNB1* expression 58. Our data similarly revealed that DSF alone, especially at low concentration, had negligible effect on the activation of cGAS-STING pathway, while a combination treatment of DSF and Cu (II) at extremely low dose significantly and consistently triggered this pathway across multiple cancer cell lines, highlighting the critical role of Cu addition in activating innate immunity. Moreover, excessive Cu can bind to mitochondrial proteins and disrupt the electron transport chain, thereby releasing mitochondrial-derived ROS and inducing DNA damage 59. However, our findings indicated that Cu alone was insufficient to effectively activate *IFNB1* expression *in vitro*, probably due to the limited capacity of low Cu concentrations to significantly elevate ROS levels.

As a copper ionophore, DSF promotes the intracellular transport of Cu 24. Upon DSF/Cu treatment, two mechanisms may contribute to excessive ROS production: firstly, DSF and Cu, due to their redox properties, directly generate high levels of ROS through redox reactions in the short term; secondly, excessive copper accumulation in mitochondria induces mitochondrial-derived ROS release and cuproptosis, a process that occurs later and persists longer. Consequently, the DSF/Cu combination not only generates higher levels of ROS but also maintains their elevation, leading to sustained oxidative stress, DNA damage, and activation of innate immunity 34. Nevertheless, since the Cu content in *in vitro* cultured cell lines is relatively low 60, administering low concentrations of DSF alone may not effectively induce excessive ROS production, rendering it insufficient to cause DNA damage or activate innate immunity. Other studies have reported that DSF treatment alone can effectively enhance *in vivo* anti-cancer immunity, which are attributed to mechanisms independent of Cu, such as inducing PD-L1 up-regulation by suppressing DNMT1 expression and activity 61 or inhibiting the accumulation and polarization of tumor-suppressive macrophages by targeting FROUNT 23. However, Cu levels are significantly higher in malignant tissues than normal tissues and* in vitro* cell lines 60, 62. It holds possibility that the originally existed Cu within tumor may also contribute to the immunomodulatory effects of DSF treatment *in vivo*. In brief, our study empathizes that DSF-induced activation of innate immunity in *in vitro* cancer cells is Cu-dependent. Further *in vivo* studies are needed to compare the efficacy of DSF alone and in combination with Cu in activating innate immunity, suppressing tumor growth and metastasis, as well as enhancing response to ICBs therapy.

Notably, two recent studies have suggested that DSF serves as the inhibitors of GSDMD and RNF15 to suppress STING-dependent pathway in innate immune cells, showing potential in ameliorating lung ischemia/reperfusion and autoimmune diseases 63, 64. However, the present study did not observe definite inhibitory effect of DSF on innate immunity in cancer cells, suggesting that DSF alone or in combination with Cu may exhibit different therapeutic efficacies depending on the disease context. These observations warrant further investigation into the detailed mechanisms underlying DSF's actions in various disease settings.

Mechanistically, considering the reproducibility across multiple cancer cell lines, we propose that DSF/Cu activates cGAS-STING signaling pathway via a common way, that is, by generating excessive ROS to induce DNA damage and cytosolic dsDNA leakage. Through eliminating ROS by NAC, the activating effect was perfectly reversed, which validated our initial hypothesis. In addition, the formation of bis (diethyldithiocarbamate)-copper (CuET) from dithiocarb (DTC, a reduced metabolite of disulfiram) and Cu^2+^ has been recognized as another important mechanism underlying the anti-cancer effect of DSF/Cu 22, 65. CuET exhibited preferential accumulation in tumors treated with DSF/Cu, and blocked the P97-NPL4 pathway to induce proteasome inhibition and cell stress, thereby leading to tumor suppression 22. As P97-NPL4 pathway has been suggested to be closely associated with inhibition of RIG-I-mediated innate immune signaling pathway 66 and maintenance of regulatory T cell development 67, it is possible that DSF/Cu targets P97-NPL4 pathway to activate innate immunity and enhance anti-cancer immunity. Moreover, although previous studies have reported that DSF negatively regulates innate immunity in non-malignant diseases 63, 64, it cannot be ruled out that DSF/Cu affects immune cells in tumor microenvironment by modulating cGAS-STING pathway or other mechanisms, thus promoting anti-cancer immunity. Further studies are required to clarify these interactions and provide a comprehensive understanding of the immunomodulatory effects of DSF/Cu.

**Figure 7:**
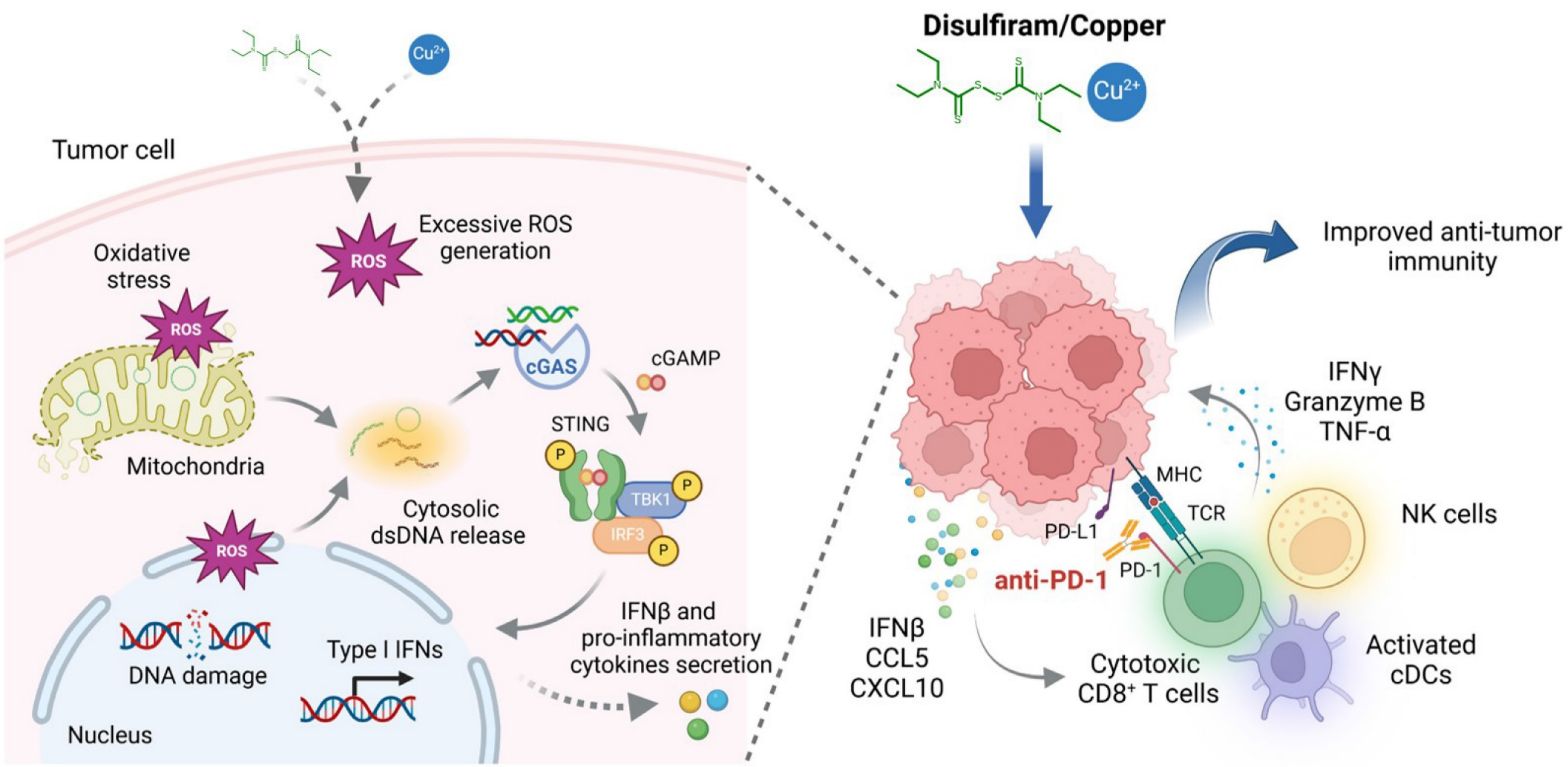
** A schematic model illustrating the mechanisms by which DSF/Cu activates innate immune signaling pathway to enhance anti-tumor immunity and potentiate therapeutic responses to PD-1 checkpoint blockade (created with BioRender.com).** DSF/Cu induces oxidative stress and DNA damage in tumor cells by generating excessive ROS. This process leads to the release of cytosolic dsDNA, which robustly activates the cancer cell-intrinsic cGAS-STING-dependent innate immune signaling pathway. Consequently, tumor cells produce IFNβ and pro-inflammatory chemokines, such as CCL5 and CXCL10, which recruit conventional dendritic cells (cDCs), CD8^+^ cytotoxic lymphocytes, and natural killer (NK) cells into the tumor microenvironment (TME). This infiltration converts the TME into an inflamed or "hot" immunophenotype, enhancing sensitivity to immune checkpoint blockade therapies. Therefore, the combination of DSF/Cu with anti-PD-1 antibodies significantly enhances immune responses, demonstrating superior therapeutic efficacy in suppressing tumor growth.

In conclusion, the present study elucidates a novel mechanism by which DSF/Cu enhances anti-cancer immunity. This effect is mediated by the activation of cGAS-STING-dependent cancer cell-intrinsic innate immune signaling pathway, driven by excessive ROS generation that induces DNA damage and cytosolic dsDNA release (Fig. 7). We also determine therapeutic efficacy of DSF/Cu in murine tumor models and demonstrate its potential to improve anti-tumor responses to PD-1 checkpoint blockade. Given that DSF and copper supplements are already under clinical evaluation for cancer therapy, we expect that our findings provide valuable insights that help facilitate the clinical translation of DSF repurposing in cancer treatment.

## Supplementary Material

Supplementary figures and tables.

## Abbreviations

ALT: alanine transaminase
AST: aspartate aminotransferase
BUN: blood urea nitrogen
CRE: creatinine
Cu: copper
dsDNA: double-stranded DNA
DSF: disulfiram
ELISA: enzyme-linked immunosorbent assay
H&E: hematoxylin-eosin
ICBs: immune checkpoint blockades
IF: immunofluorescence
IFN: interferon
IHC: immunohistochemical
IRF3: interferon regulatory factor 3
NAC: N-acetylcysteine
qRT-PCR: quantitative reverse transcription PCR
ROS: reactive oxygen species
siRNAs: short interfering RNAs
STAT1: signal transducer and activator of transcription 1
STING: stimulator of interferon genes
TBK1: tank-binding kinase 1
TME: tumor microenvironment
