# Supplementary material for: Disulfiram/copper triggers cGAS-STING innate immunity pathway via ROS-induced DNA damage that potentiates antitumor response to PD-1 checkpoint blockade
Source: Int J Biol Sci. 2025 Feb 3;21(4):1730–48. doi: 10.7150/ijbs.105575 (PMC11844283; doi:10.7150/ijbs.105575)
Supplement: Supplementary file 1 — Supplementary figures and tables. [file ijbsv21p1730s1.pdf]

Supplementary Data

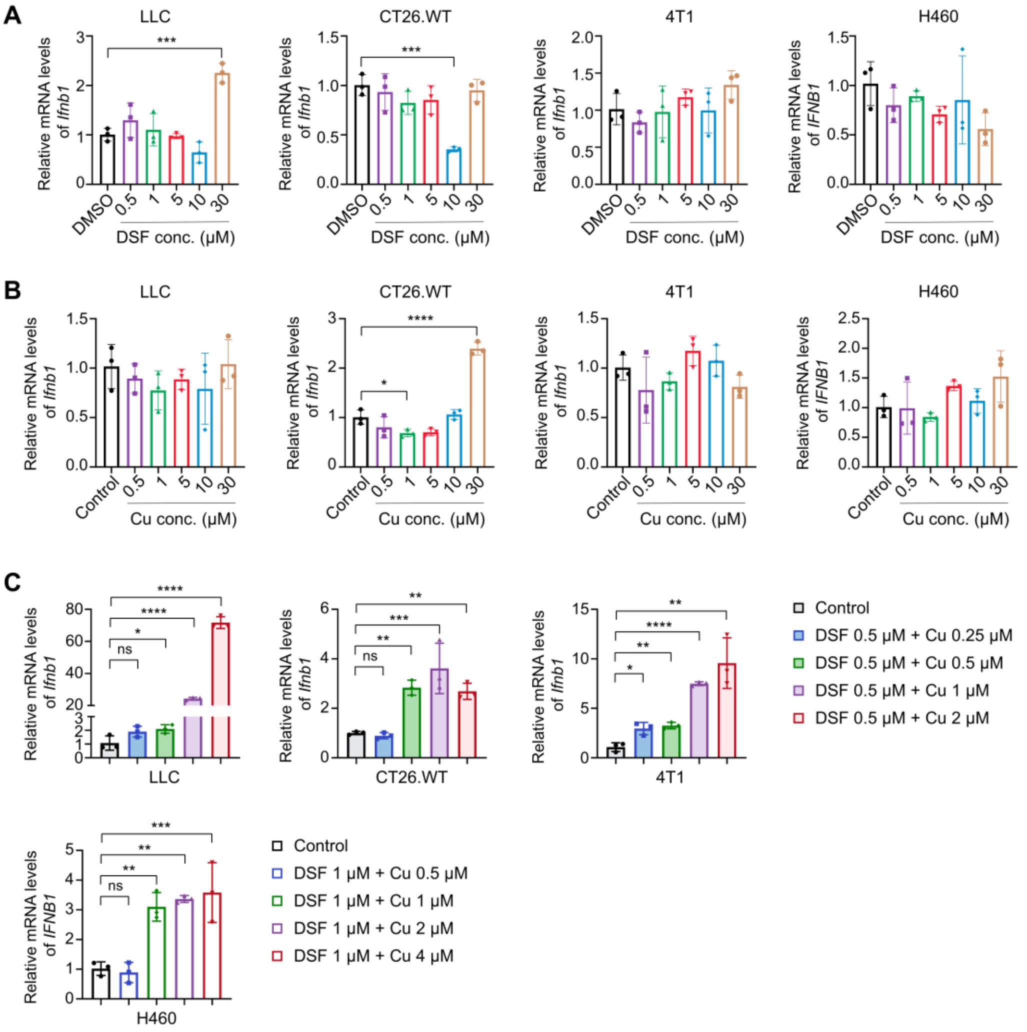

Figure S1. Cu is essential for DSF activating cancer cell- intrinsic innate immunity at low concentrations, related to Fig. 1. (**A-B**) Cells were treated with the indicated concentrations of DSF (A) or $CuCl_2$ (B) for 24 h individually. Control groups were synchronously treated with the same volume of DMSO or water. qRT-PCR was performed to detect mRNA levels of IFNB1 in the indicated cells. (**C**) Cells were treated with the indicated concentrations of DSF/$CuCl_2$ in combination (at different molar ratios) for 24 h. qRT-PCR was performed to detect mRNA levels of IFNB1 in the indicated cells. All data are shown as mean ± SD of independent biological replicates, and p values were determined by one-way ANOVA followed by Tukey's multiple comparisons test.

****p < 0.0001, ***p < 0.001, **p < 0.01, *p < 0.05; ns, not significant.

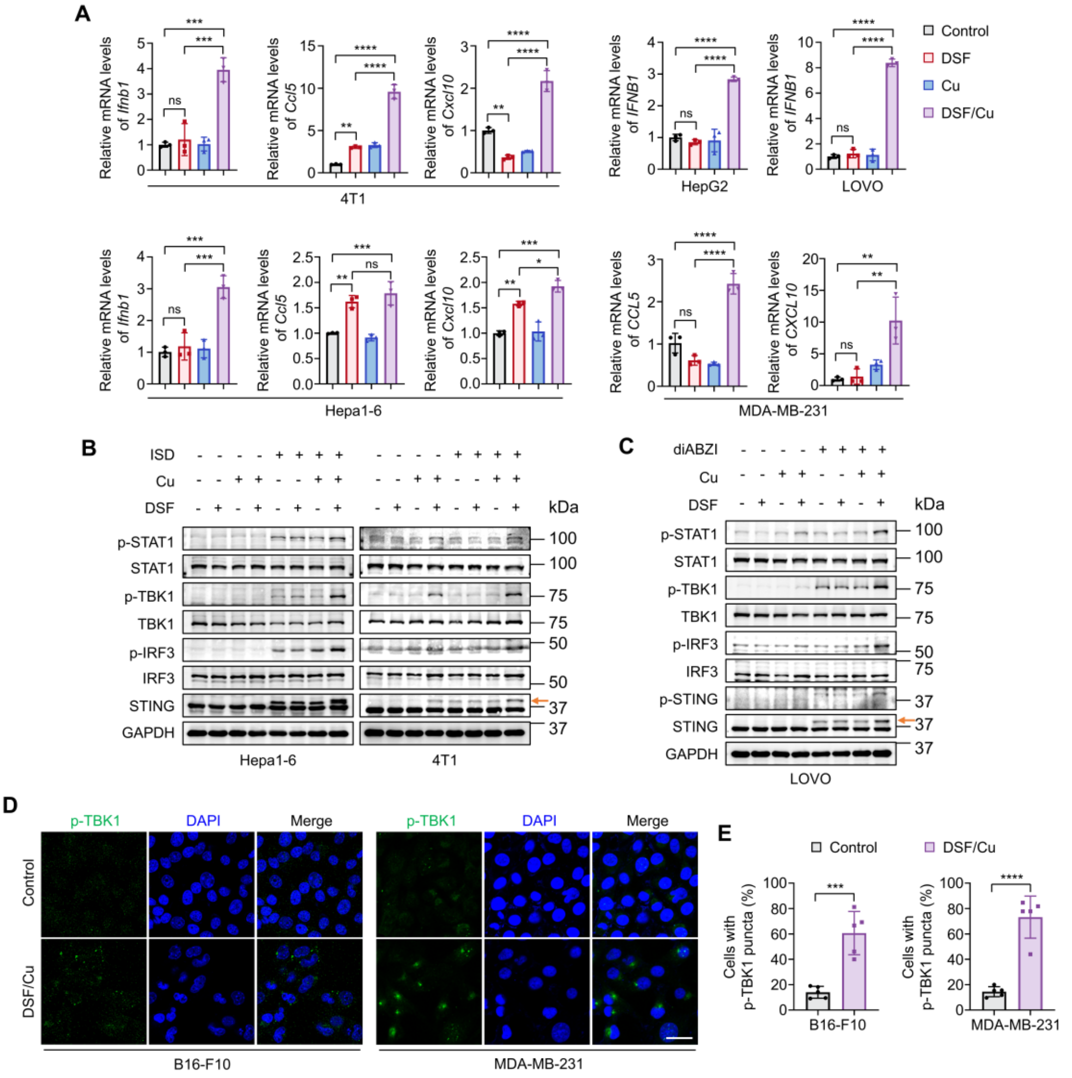

Figure S2. DSF/Cu activates cGAS-STING-dependent cancer-cell intrinsic innate immunity, related to Fig. 1. Cells were treated with DSF (1 μM for HepG2 and LOVO, 0.5 μM for others) and $CuCl_2$ (1 μM for HepG2 and LOVO, 0.5 μM for others), individually or in combination (at 1:1 molar ratio), for 24 h. Control groups were synchronously treated with the same volume of DMSO or water. (**A**) qRT-PCR was performed to detect mRNA levels of IFNB1, CCL5 and CXCL10 in the indicated cells. (**B-C**) Immunoblotting analysis of the indicated proteins in cancer cells treated with DSF and/or $CuCl_2$, followed by 1 μg/mL ISD stimulation for 4 h (B) or 5 μM diABZI stimulation

(C) for 2 h. Orange arrow points to the band of phosphorylated STING. (**D**) Representative fluorescence images of DAPI (blue) and p-TBK1 (green) staining in B16-F10 and MDA-MB-231 cells with the indicated treatments. Scale bar, 30 μm. (**E**) Quantification of the percentage of cells with one or more p-TBK1 puncta. All data are shown as mean ± SD of independent biological replicates, and p values were determined by unpaired Student's t test (for two groups data) or one-way ANOVA followed by Tukey's multiple comparisons test (for four groups data). ****p < 0.0001, ***p < 0.001, **p < 0.01, *p < 0.05; ns, not significant.

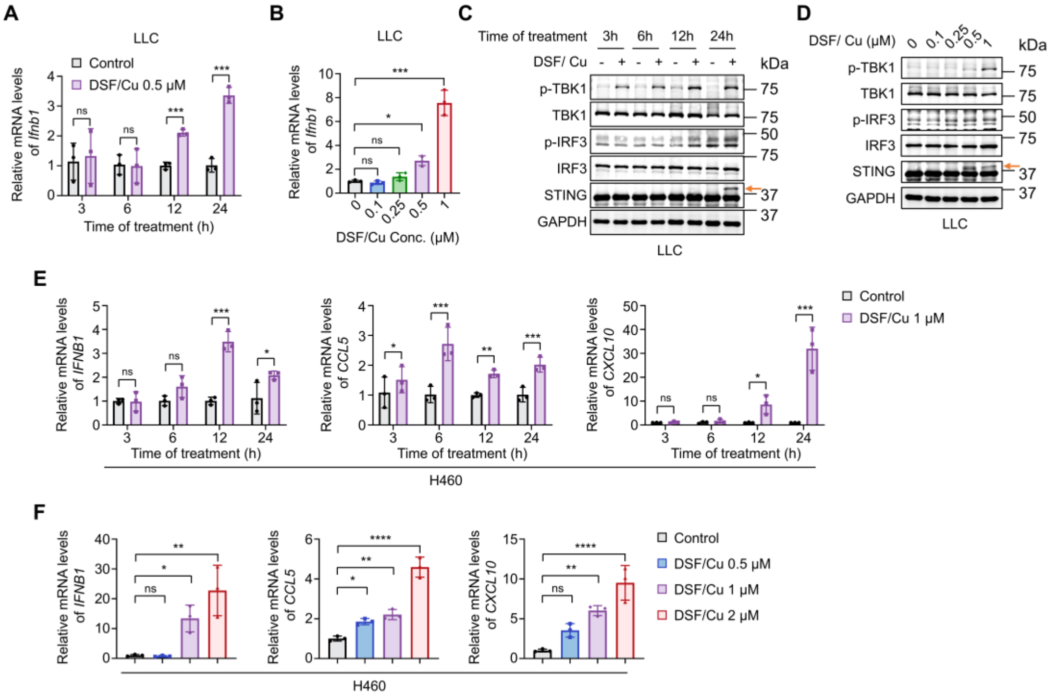

Figure S3. DSF/Cu activates cell-intrinsic innate immunity in a time- and dose-dependent manner, related to Fig. 1. LLC and H460 cells were treated with DSF/$CuCl_2$ (0.5 μM for LLC and 1 μM for H460, at 1:1 molar ratio) for the indicated times (**A**, **C,** and **E**) or treated with the indicated concentrations of DSF/$CuCl_2$ for 24 h (**B**, **D,** and **F**). (**A**, **B**) **q**RT-PCR was performed to detect mRNA levels of IFNB1 in LLC cells treated as indicated. (**C**, **D**) Immunoblotting analysis of the

indicated proteins in LLC cells treated as indicated. (**E**, **F**) **q**RT-PCR was performed to detect mRNA levels of IFNB1, CCL5 and CXCL10 in H460 cells treated as indicated. All data are shown as mean ± SD of independent biological replicates, and p values were determined by unpaired Student's t test (for two groups data) or one-way ANOVA followed by Tukey's multiple comparisons test (for four groups data). ****$p < 0.0001$, ***$p < 0.001$, **$p < 0.01$, *$p < 0.05$; ns, not significant.

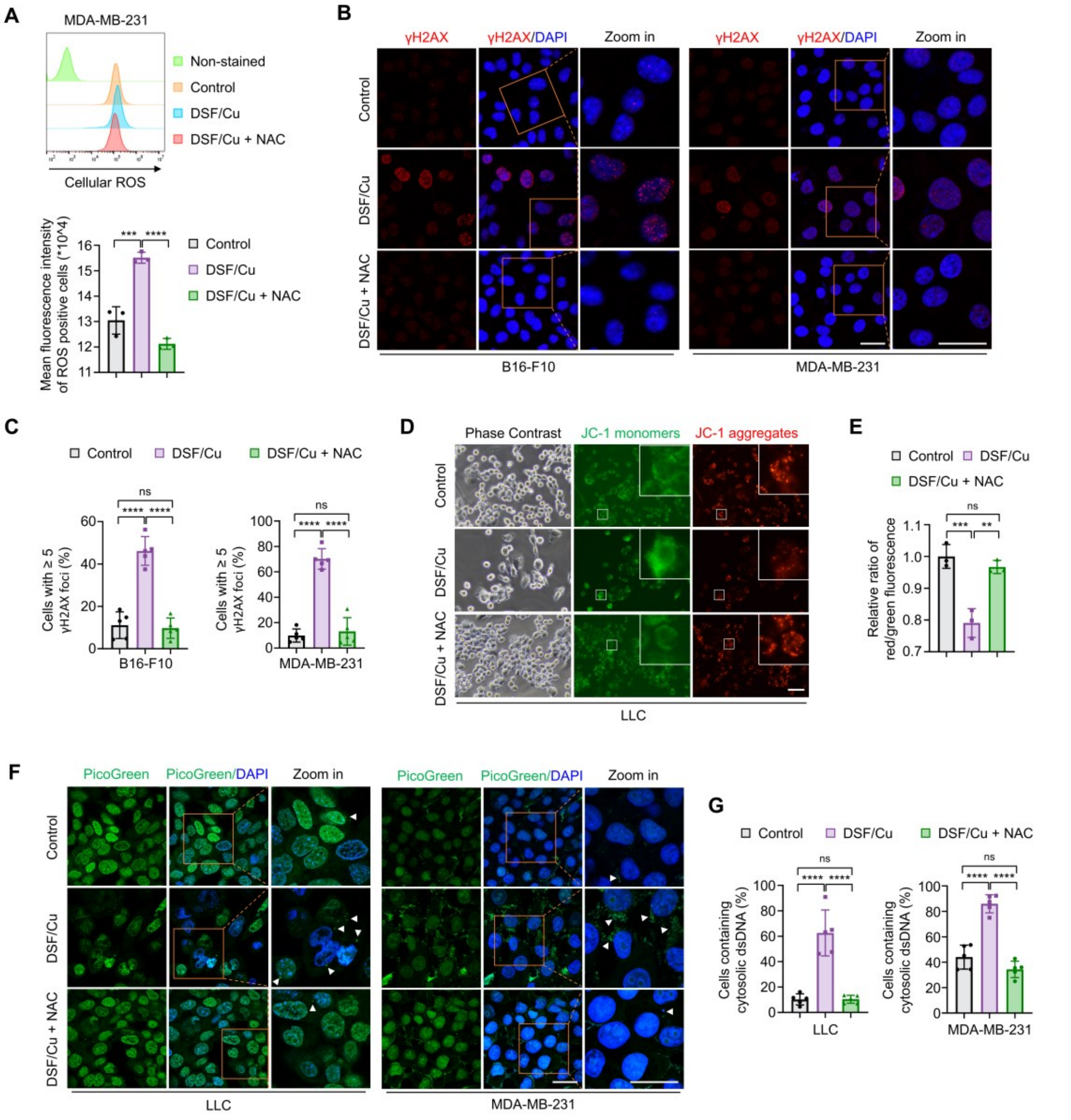

Figure S4. DSF/Cu induces nuclear and mitochondrial DNA damage and dsDNA leakage in a ROS-dependent manner, related to Fig. 2. Cells were treated with 0.5 μM DSF/Cu alone or in

combination with 2 mM NAC for 24 h. (**A**) Flow cytometry showing cellular ROS levels of MDA-MB-231 cells treated as indicated (upper panel). Lower panel showing the quantitative analysis of ROS levels based on the mean intensity of the CellROX® Deep Red fluorescence on flow cytometer. (**B**) Representative images of γH2AX foci (red) and DAPI nuclear staining (blue) in B16-F10 and MDA-MB-231 cells treated as indicated. Scale bars, 30 μm. (**C**) Quantification of the percentage of cells with ≥5 γH2AX foci per nucleus in the indicated groups. (**D**) Representative images of JC-1 fluorescence probe-stained mitochondrial membrane potential changes in cells treated as indicated. Green fluorescence showed JC-1 as monomers when mitochondrial membrane potential was in a low level, while red fluorescence showed JC-1 aggregating in mitochondrial matrix when mitochondrial membrane potential was in a high level. Scale bar, 100 μm. (**E**) Quantitative analysis of the relative ratios of JC-1 red/green fluorescence in the indicated groups. (**F**) Representative images of PicoGreen staining (green) and DAPI nuclear staining (blue) in LLC and MDA-MB-231 cells treated as indicated. Scale bars, 30 μm. (**G**) Quantification of the percentage of cells containing cytosolic dsDNA foci. All data are presented as mean ± SD of independent biological replicates, and p values were determined by one-way ANOVA followed by Tukey's multiple comparisons test. ****$p < 0.0001$, ***$p < 0.001$, **$p < 0.01$; ns, not significant.

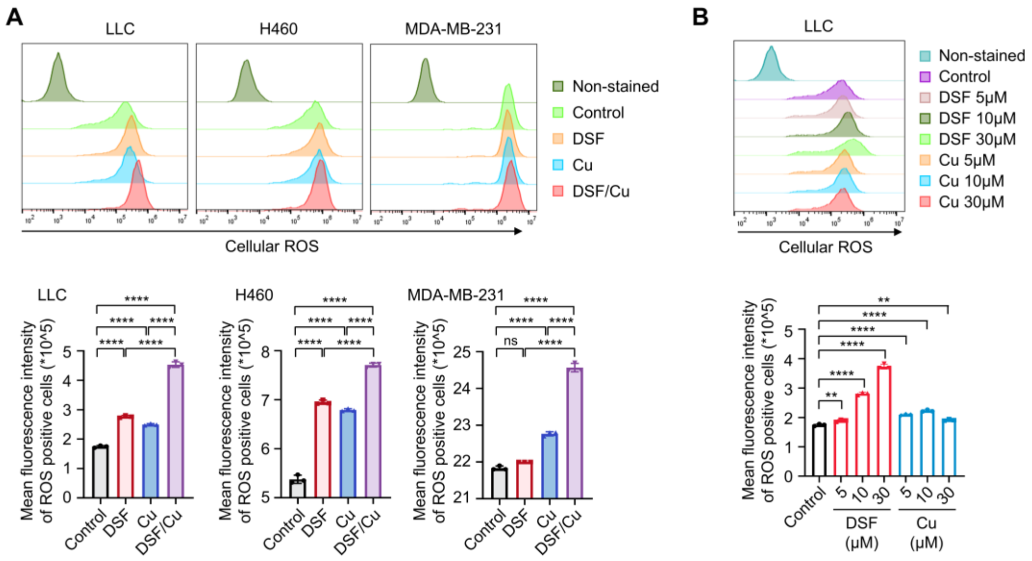

Figure S5. DSF/Cu in combination induces higher ROS level than DSF or Cu individually, related to Fig. 2. (**A**) The indicated cells were treated with DSF/Cu (0.5 µM for LLC and 1 µM for others, at 1:1 molar ratio) alone or in combination for 24 h. Upper panel showed representative flow cytometry results of cellular ROS levels of cells treated as indicated. Lower panel indicated the quantitative analysis of ROS levels based on the mean intensity of the DCFH fluorescence on flow cytometer. (**B**) LLC cells were treated with the indicated concentrations of DSF or $CuCl_2$ for 24 h individually. Representative flow cytometry result (upper panel) and the quantitative analysis (lower panel) of cellular ROS levels based on the mean intensity of the DCFH fluorescence on flow cytometer. All data are presented as mean ± SD of independent biological replicates, and p values were determined by one-way ANOVA followed by Tukey's multiple comparisons test. ****$p < 0.0001$, ***$p < 0.001$, **$p < 0.01$; ns, not significant.

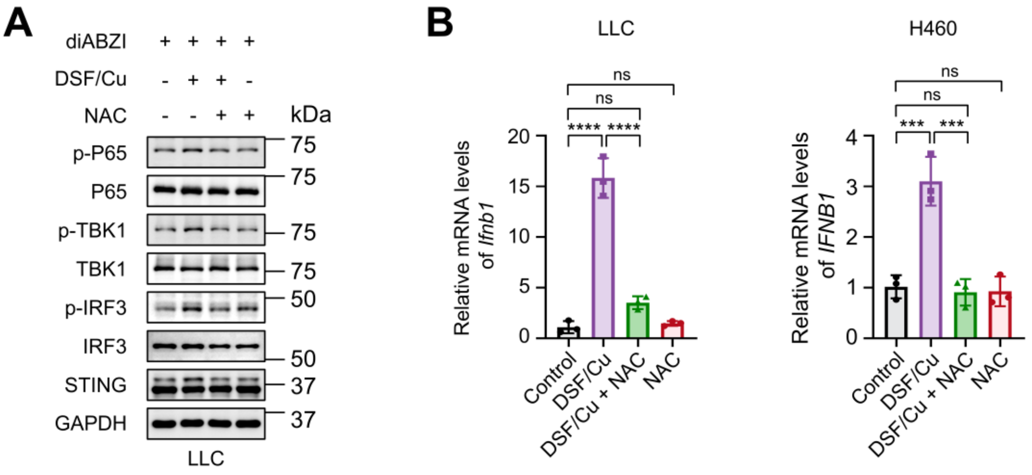

Figure S6. NAC when used alone causes no significant effect on innate immunity in cancer cells, related to Fig. 3. (**A**) LLC cells were treated with 0.5 µM DSF/Cu (at 1:1 molar ratio) or 2 mM NAC for 24 h, followed by 2.5 µM diABZI stimulation for 2 h. Immunoblotting analysis of the proteins in cells treated as indicated. (**B**) Cells were treated with DSF/Cu (0.5 µM for LLC and 1 µM for H460, at 1:1 molar ratio) or 2 mM NAC alone or in combination for 24 h. qRT-PCR was performed to detect mRNA levels of IFNB1 in indicated cells. All data are presented as mean ± SD of independent biological replicates, and p values were determined by one-way ANOVA followed by Tukey's multiple comparisons test. ****p < 0.0001, ***p < 0.001; ns, not significant.

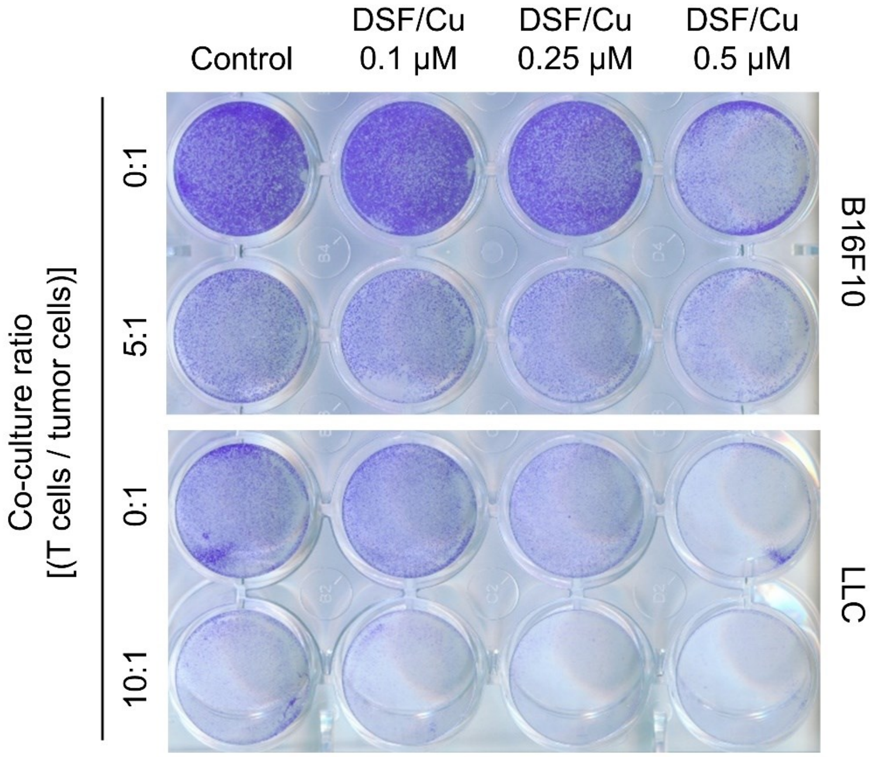

Figure S7. DSF/Cu promotes T cell-mediated tumor cell killing *in vitro*. *In vitro* T cell killing assay for pre-activated T cells co-cultured with LLC or B16-F10 cells treated as indicated for 2 days. Surviving tumor cells were visualized by crystal violet staining.

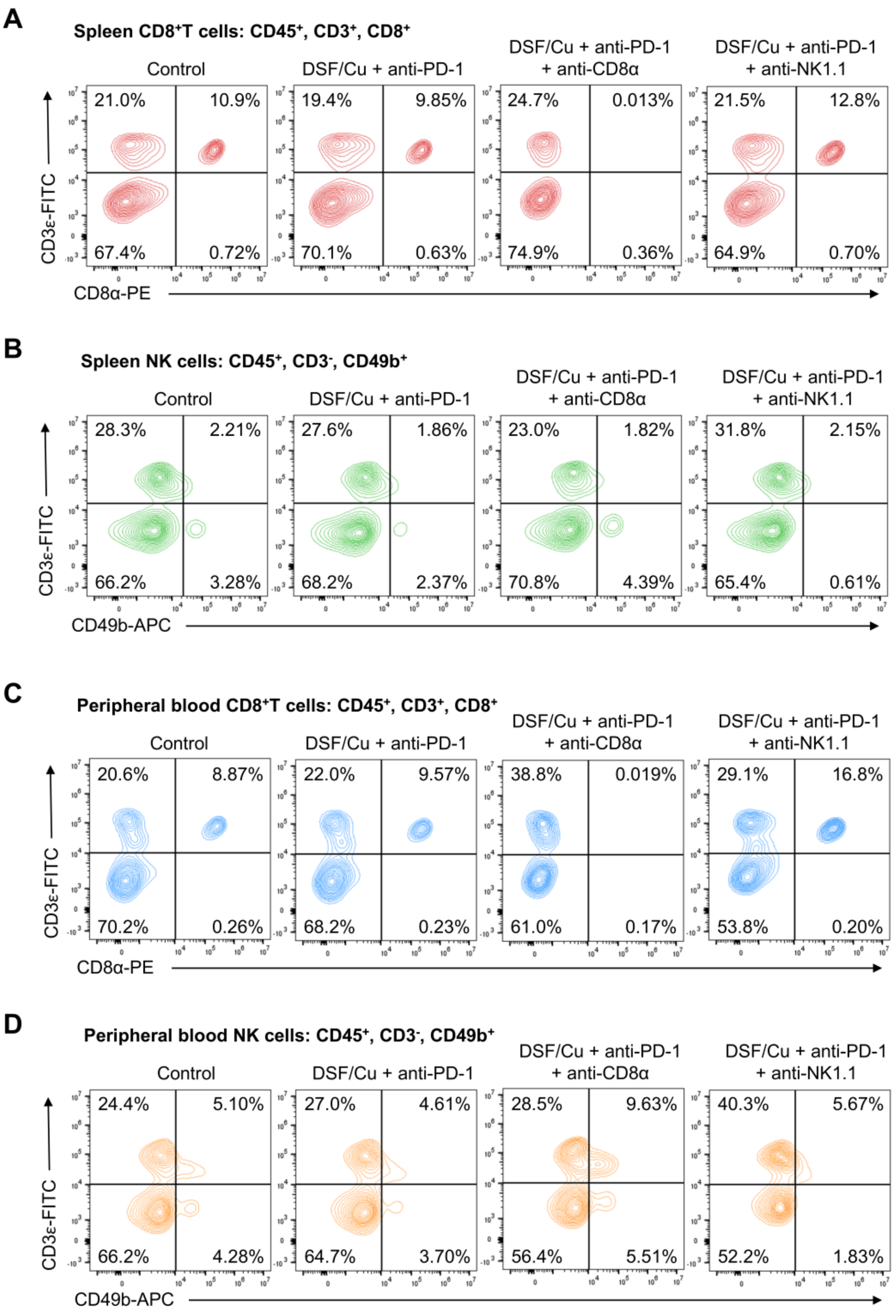

Figure S8. Analysis of CD8$^+$ T and NK cells depletion *in vivo*, related to Fig. 6. Flow cytometry plots show CD8$^+$ T and NK cell populations from the spleen and peripheral blood of LLC-tumor bearing mice after treatments with the indicated chemicals and antibodies. (**A-B**) Flow cytometry

analysis of CD45^{+} CD3^{+} CD8^{+} T cell (A) or CD45^{+} CD3^{-} CD49b^{+} NK cell (B) population from the spleen. (**C-D**) Flow cytometry analysis of CD45^{+} CD3^{+} CD8^{+} T cell (C) or CD45^{+} CD3^{-} CD49b^{+} NK cell (D) population from the peripheral blood.

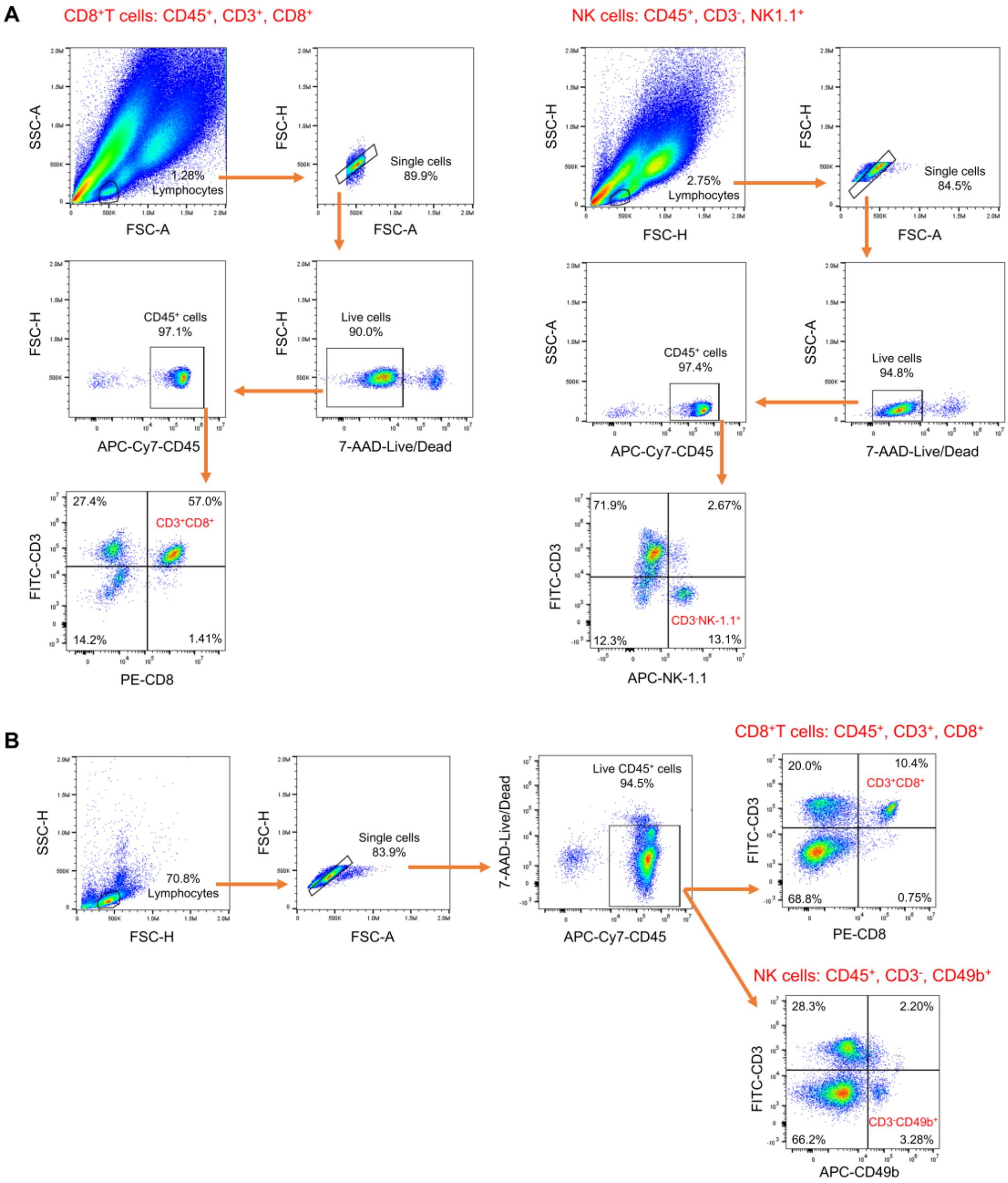

Figure S9. The flow cemetery gating strategies for CD8^{+} T and NK cells. (**A**) Representative flow cytometry gating strategies utilized for sorting CD45^{+} CD3^{+} CD8^{+} T cells (left panel) and CD45^{+} CD3^{-} NK1.1^{+} NK cells (right panel) populations in sing-cell suspension of LLC tumors. (**B**)

Representative flow cytometry gating strategies utilized for sorting $CD45^+$ $CD3^+$ $CD8^+$ T cells and $CD45^+$ $CD3^-$ $CD49b^+$ NK cells populations in sing-cell suspension of mouse spleen and peripheral blood.

Supplementary Table S1. The antibodies used in this study

Antibodies	Catalog#	Source	Application*
Anti-phospho-STING (Ser366)	19781	Cell Signaling Technology	WB
Anti-STING	19851-1-AP	Proteintech	WB
Anti-phospho-IRF-3 (Ser396)	29047	Cell Signaling Technology	WB
Anti-IRF3	80519-1-RR	Proteintech	WB
Anti-phospho-TBK1 (Ser172)	5483	Cell Signaling Technology	WB, IF
Anti-TBK1	38066	Cell Signaling Technology	WB
Anti-phospho-STAT1 (Tyr701)	9167	Cell Signaling Technology	WB
Anti-STAT1	10144-2-AP	Proteintech	WB
Anti-phospho-Histone H2A.X (Ser139)	05-636	Sigma-Aldrich	WB, IF
Anti-GAPDH antibody	AB0037	Abways	WB
Anti-Ki-67	ab15580	Abcam	IHC, IF
Anti-CD8α	ab217344	Abcam	IF
Anti-CD3	ab231775	Abcam	IF
HRP-conjugated Goat anti-Rabbit IgG H&L	ZB-2301	ZSGB-BIO	WB
HRP-conjugated Goat anti-Mouse IgG H&L	ZB-2305	ZSGB-BIO	WB
Alexa Fluor 488-labeled anti-rabbit secondary antibody	A-11008	Thermo Fisher Scientific	IF
Alexa Fluor 555-labeled anti-mouse secondary antibody	A-21422	Thermo Fisher Scientific	IF
APC/Cyanine7 anti-mouse CD45 antibody	103116	BioLegend	FC
FITC anti-mouse CD3 antibody	100203	BioLegend	FC
PE anti-mouse CD8α antibody	100707	BioLegend	FC
APC anti-mouse NK-1.1 antibody	108709	BioLegend	FC
APC anti-mouse CD49b antibody	108909	BioLegend	FC
TruStain FcX™ PLUS (anti-mouse	156603	BioLegend	FC

CD16/32) antibody			
Anti-mouse PD-1 (CD279)-InVivo	A2122	Selleck	IVT
Rat IgG2a isotype control-InVivo	A2123	Selleck	IVT
Anti-mouse CD8α-InVivo	A2102	Selleck	IVT
Anti-mouse NK1.1-InVivo	A2114	Selleck	IVT

* Western Blot (WB), Immunofluorescence (IF), immunohistochemical (IHC), Flow cytometry (FC), In vivo treatment (IVT).

Supplementary Table S2. The primer sequences used in this study for qRT-PCR

**Genes**	**Forward (5'-3')**	**Reverse (3'-5')**
human *IFNB1*	CAACAAGTGTCTCCTCCAAAT	TCTCCTCAGGGATGTCAAAG
human *CCL5*	CCTGCTGCTTTGCCTACATTGC	ACACACTTGGCGGTTCTTTCGG
human *CXCL10*	GTGGCATTCAAGGAGTACCTC	TGATGGCCTTCGATTCTGGATT
human *CGAS*	AGGAAGCAACTACGACTAAAGCC	CGATGTGAGAGAAGGATAGCCG
human *STING1*	GGGCTGGCATGGTCATATTA	TACTCAGGTTATCAGGCACC
human *ACTB*	CCTTGCACATGCCGGAG	GCACAGAGCCTCGCCTT
mouse *Ifnb1*	TCCGAGCAGAGATCTTCAGGAA	TGCAACCACCACTCATTCTGAG
mouse *Ccl5*	GCTGCTTTGCCTACCTCTCC	TCGAGTGACAAACACGACTGC
mouse *Cxcl10*	CCAAGTGCTGCCGTCATTTTC	GGCTCGCAGGGATGATTTCAA
mouse *Actb*	CATTGCTGACAGGATGCAGAAGG	TGCTGGAAGGTGGACAGTGAGG

Supplementary Table S3. siRNA sequences

**siRNAs**	**Sequence (5'-3')**
si-NC	UAAGGCUAUGAAGAGAUAC
si-CGAS #1	GAAUUCAACUAGAAGAAUA
si-CGAS #2	GGAAGCAACUACGACUAAA
si-STING1 #1	UGUUGCUGCUGUCCAUCUA
si-STING1 #2	GGUCAUAUUACAUCGGAUA
